# Detecting extracellular G4 DNA motifs in biofilms through energy transfer between DNA-binding dyes TOTO™-1 and SYTO™60

**DOI:** 10.1093/nar/gkag712

**Published:** 2026-07-20

**Authors:** Line Mørkholt Lund, Gabriel Antonio Salvador Minero, Julie Kaysen, Rikke Louise Meyer, Victoria Birkedal

**Affiliations:** Interdisciplinary Nanoscience Center (iNANO), Aarhus University, 8000 Aarhus, Denmark; Department of Chemistry, Aarhus University, 8000 Aarhus, Denmark; Interdisciplinary Nanoscience Center (iNANO), Aarhus University, 8000 Aarhus, Denmark; Department of Biology, Aarhus University, 8000 Aarhus, Denmark; Interdisciplinary Nanoscience Center (iNANO), Aarhus University, 8000 Aarhus, Denmark; Department of Chemistry, Aarhus University, 8000 Aarhus, Denmark; Interdisciplinary Nanoscience Center (iNANO), Aarhus University, 8000 Aarhus, Denmark; Department of Biology, Aarhus University, 8000 Aarhus, Denmark; Interdisciplinary Nanoscience Center (iNANO), Aarhus University, 8000 Aarhus, Denmark; Department of Chemistry, Aarhus University, 8000 Aarhus, Denmark

## Abstract

Secondary DNA structures in extracellular DNA are important for the function and resilience of bacterial biofilms. Non-canonical DNA structures are, however, challenging to localize and characterize. We demonstrate here a FRET-based approach to detect G-quadruplexes (G4s) *in vitro* and *in situ* in the extracellular matrix of bacterial biofilms by combining the DNA-binding dyes TOTO™-1 and SYTO™60. Upon addition of the dyes and excitation of TOTO™-1, we observed strong energy transfer (FRET) between the two dyes in synthetic G4 DNA structures. Subsequently, we demonstrate the application of FRET to visualize G4 DNA in the extracellular matrix of biofilms and that these motifs are enriched upon treatment with DNase I. Our work shows a novel and robust method for analysis of G4 DNA motifs in complex biological samples using FRET-pair dyes with high affinity toward G4s.

## Graphical Abstract

**Graphical Abstract:**
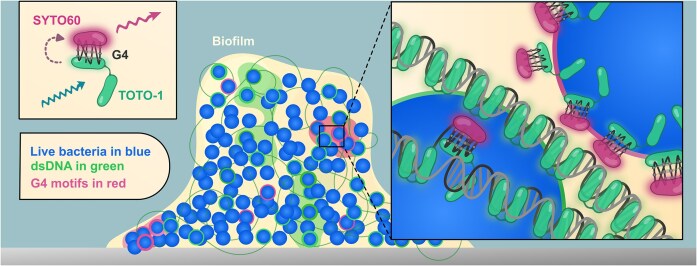

## Introduction

G-quadruplexes (G4s) are non-canonical DNA structures composed of several consecutive π–π stacked guanine tetrads connected via Hoogsteen hydrogen bonding and stabilized by a monovalent cation [1]. Unlike double-stranded DNA (dsDNA), G4s display high structural diversity. They can be categorized by strand orientation into parallel, hybrid, and antiparallel geometries. G4s may fold intramolecular structures from one single-strand of DNA or RNA, or intermolecular structures from two or four single strands, as well as higher-order intermolecular G4 wires under certain conditions [2]. The formation of G4s plays an important role in transcriptional and translational regulation of genes, DNA replication, genome stability, and oncogene expression in eukaryotic genomes [1].

In microbiology, the roles of G4 DNA and G4 RNA are starting to be explored [3–5], and novel functions are being discovered for G4s in extracellular DNA (eDNA). Recently, G4s were found in the extracellular matrix of *Pseudomonas aeruginosa* [6] and *Staphylococcus epidermidis* [7] biofilms: aggregated microbial communities encased in a protective extracellular matrix. Here, the G4s were either associated with the bacterial cell envelope or formed part of an eDNA network connecting bacteria across millimeter distances. In infections, extracellular G4s are also found in eDNA originating from immune cells (neutrophils) that release chromatin to form a net-like extracellular trap to immobilize and encapsulate the infection [8].

eDNA is important for the biofilm’s structural integrity. While the extracellular matrix also contains polysaccharides and proteins, it is the eDNA that primarily impacts the viscoelastic properties of biofilms [9]. The discovery of extracellular G4s has added several intriguing functions to eDNA in biofilms. G4s were reported to promote viscoelasticity [6], and since they resist degradation by mammalian DNase [7], they might contribute to the resilience of biofilm infections [10]. Furthermore, extracellular G4s can bind hemin to form a DNAzyme with peroxidase-like catalytic activity, which bacteria can use for extracellular electron transfer to sustain metabolic activity under oxygen-limiting conditions [11]. Hence, G4s provide several functional properties to biofilms that need further elucidation.

The presence and function of G4s in various biological processes have stimulated the development of tools to visualize G4 DNA in cells. The use of G4-antibodies for immunolabeling of fixed cells for G4 imaging by fluorescence is a key approach but not suitable for use in live cells [12, 13]. Recently, a high-affinity G4 nanobody has been developed for live-cell G4 imaging [14]. There are also several fluorescent probes that have a preference for G4, which have been successful in detecting and studying pure G4s as well as G4 DNA in cells [15]. Such probes overcome some of the limitations of antibodies in terms of live-cell imaging and are advantageous because of their small size that allows detection of closely spaced G4s. For example, N-methyl mesoporphyrin (NMM) is a light-up probe for G4s with parallel topology [16]. However, NMM penetrates cells and is therefore not suited to probe extracellular G4s specifically. Thioflavin T, another light-up probe used for G4 detection [17], allows identification of a broad range of G4 motifs and topologies. A derivative of Thioflavin T, IMT, has been successfully used for G4 imaging in live cells [18], and a fluorophore-conjugated pyridostatin ligand has achieved single-molecule G4 imaging [19]. So far, extracellular G4s have been identified in biofilms [6, 7] using immunolabeling with the antibodies 1H6 [13] and BG4 [12] and by visualization of hemin/G4 peroxidase activity by tyramide signal amplification [20, 21].

A challenge for G4 DNA motif detection in complex biological samples is the presence and abundance of other biomolecules and other DNA structures. The G4 might only constitute a small fraction of the total DNA in such samples. These conditions put a very strong requirement on the specificity of G4 probes when G4 structures are surrounded by large amounts of dsDNA. Such a high specificity is difficult to achieve as many G4 fluorescent light-up probes also bind to single- and dsDNA to some extent. A way to circumvent this difficulty is the use of environmentally sensitive probes that have different fluorescent lifetimes when bound to dsDNA versus G4s. These probes, combined with fluorescence lifetime imaging, have been successfully used to detect DNA and RNA G4s in cells [22, 23]. Another way to increase detection selectivity is to use two-color probes to detect G4s through energy transfer efficiency between the two dye molecules that strongly depend on their distance. G4s bind cyanine stains such as thiazole orange (TO) [24], and FRET sensitization of TO templated by G4 DNA using a G4-specific binder as donor was previously reported [25]. For G4 detection in the extracellular matrix of biofilms, at least one of the dyes should not be cell-permeable, and we therefore wondered if extracellular G4s can be visualized using the SYTO™60/TOTO™-1 couple applied for live/dead bacterial staining [26].

TOTO™-1 is a bright and efficient light-up probe commonly used in DNA staining [27], not highly cell-permeable, and used in combination with the hydrophobic cyanine-based molecule SYTO™60 for dead/live bacterial staining [28, 29]. The dimeric TOTO™-1 and SYTO™60 (both derivatives of TO) have spectral properties compatible with the possibility of FRET and may have different affinities for double-stranded and G4 DNA. We hypothesize that this FRET effect can give a readout for G4 motif detection. Such FRET signals could provide a new approach to detecting G4s among other DNA structures in a complex sample.

In this study, we aim to develop a FRET-based approach to detect G4s *in vitro* and *in situ* in the extracellular matrix of bacterial biofilms by combining the DNA-binding dyes TOTO™-1 and SYTO™60. We first examined and optimized the FRET readout using a short G4 DNA motif as a benchmark for our studies. We determined dissociation constants (*K*_D_) of the DNA-binding dyes TOTO™-1 and SYTO™60 using pre-folded single-stranded, double-stranded, and G4 DNA substrates. Based on the difference between the affinities of the dyes to different DNA substrates, we proceeded with quantifying the FRET signal between TOTO™-1 and SYTO™60 mediated by these DNA substrates at different dye concentrations and upon degradation of dsDNA by DNase I. In a next step, we investigated if this could be applied to detect extracellular G4 motifs in biofilms and developed a method for G4 fluorescence imaging using the SYTO™60/TOTO™-1 FRET pair and *S. epidermidis* biofilm models. Our results demonstrate the use of FRET for detection of extracellular G4 motifs in biofilms, which could be exploited for fast screening of complex samples.

## Materials and methods

### Oligonucleotides, nucleases, DNA-binding antibodies, and dyes

All chemicals were purchased from Sigma–Aldrich Inc. (Germany) unless otherwise specified. Oligonucleotides were purchased from Sigma–Aldrich Inc., HPLC purified by the manufacturer, hydrated in double-distilled water (ddH_2_O), and stored at −20°C unless otherwise specified. The DNA sequences are found in Table 1.

**Table 1.** DNA sequences used in this study

DNA name	DNA sequence
ssDNA	5′ - GTT GGC GTG GCA CCG GTA
dsDNA	5′ - GCA GGC GTG GCA CCG GTA ATA GGA TTA GGG TTG GGA TGC GCA AGA GAG GAC GGG
	5′ - CCC GTC CTC TCT GTC GCA TCC CAA CCC TAA TCC TAT TAC CGG TGC CAC GCC TGC
G4DNA	5′ - GA GGG T GGG TA GGG T GGG
4×G4DNA	5′ - GA GGG T GGG TA GGG T GGG GA GGG T GGG TA GGG T GGG GA GGG T GGG TA GGG T GGG GA GGG T GGG TA GGG T GGG CGT CAA CAG ACT CGA

4×G4DNA was purchased from IDT, purified by the manufacturer (standard desalting), and stored in 1 mM TE buffer (pH 8) at −20°C.

Nucleases were stored at −20°C in their storage buffers: DNase I (M0303S, 2000 units/ml, New England Biolabs) in 10 mM Tris–HCl (pH 7.5), 2 mM CaCl_2_, and 50% glycerol; DNase I (04716728001, 10 000 units/ml, Roche) in 20 mM Tris–HCl (pH 7.6), 50 mM NaCl, 2 mM CaCl_2_, 2 mM MgCl_2_, 1 mM dithioerythritol, and 50% glycerol; Micrococcal nuclease (MNase, EN0181, 300 000 units/ml, Thermo Fisher Scientific) in 20 mM Tris–HCl (pH 7.6), 50 mM NaCl, and 50% (v/v) glycerol. The New England Biolabs DNase I (up to 114 units/ml) was added to synthetic DNA in the reaction buffer (1× = 10 mM Tris–HCl, pH 7.6, 2.5 mM MgCl_2_, 0.5 mM CaCl_2_) purchased from New England Biolabs (M0303L) and incubated for 1 h at room temperature. Separately, the MNase (15 units/ml) and DNase I (500 units/ml; Roche) diluted in the universal nuclease reaction buffer [1× = 25 mM Tris-acetate (pH 6.0), 1.0 mM MgSO_4_, 6.25 mM CaCl_2_, Merck] were applied to *S. epidermidis* biofilms (37°C, 1 h) as previously discussed in the reference [7]. The pH of 1 M Tris was adjusted to pH 6.0 by 33% acetic acid (Merck). All solutions were sterile-filtered using 0.2 µm filters (83.1826.001, Fisher Scientific).

A 25 mM hemin solution was obtained by dissolving hemin (51280-1G, Merck) in 100 mM Tris with 100 mM NaOH (Merck) and 30% DMSO (pH 11). Hemin was handled and stored away from light.

The 1 mg/ml antibodies Atto488-BG4 (goat monoclonal IgG lambda) in phosphate buffer saline (PBS) with 0.02% proclin was purchased from the Absolute Antibodies as a fluorescently tagged antibody (subsequently stored in the fridge with light protection). The 1 mg/ml 1H6 antibody (mouse monoclonal IgG2bκ) in 0.1 M Tris-glycine (pH 7.4) buffer with 150 mM NaCl and 0.05% NaN_3_ was purchased from Merck. The 1 mg/ml antibody AB1 (mouse monoclonal 3519) in PBS was purchased from Abcam (aliquoted and stored at −20°C). The 2 mg/ml anti-mouse secondary antibody CF405S-AB2 (goat polyclonal IgG H + L) in PBS with 0.05% sodium azide and 50% glycerol was purchased from Merck (stored at −20°C with light protection). We used sterile-filtered 3 % bovine serum albumin (BSA heat shock fraction, pH 7, >98 %, Merck) in 1 × Pierce PBS buffer (Thermo Fisher Scientific) to prevent unspecific binding.

The DNA-binding dyes TOTO™-1 iodide, TOTO™-3 iodide, SYTO™41, and SYTO™60 in DMSO (Thermo Fisher Scientific) were aliquoted at 250 µM concentration in Milli-Q water (stored at −20°C). The G4-specific N-methyl protoporphyrin (NMP from LivChem), 20 mM in DMSO, was aliquoted at 250 µM concentration in Milli-Q water. The membrane-binding stain FM 4-64 (Thermofisher Scientific) was diluted 1 mg/mL in milliQ water. NMP and FM 4-64 were stored at 4°C. The dyes were further diluted in reaction buffer to their working concentration on the day of the experiment.

### Circular dichroism spectroscopy

DNA oligonucleotides were diluted in the assay buffer (10 mM KCl, 100 mM KCl or CsCl, and 20 mM Tris, pH 7.5) to concentrations of 2 µM (dsDNA) and 2.5 µM [single-stranded DNA (ssDNA) and G4DNA]. The DNA was thermally annealed by heating the samples to 95°C for 5 min followed by slow cooling overnight. Measurements were done using the Jasco J-810 spectropolarimeter with a sample volume of 120 µl in a 10-mm, 100-µL quartz cuvette (Hellma™) at 22°C. Circular dichroism (CD) spectra were background-corrected before analysis. Spectra were converted to molar ellipticity [θ] from the original unit of millidegrees [mdeg]:


\begin{eqnarray*}
\mathrm{Molar}\,\,\mathrm{Ellipticity}\left[ \theta \right] = \frac{{\mathrm{ CD}}}{{10 \cdot C \cdot L}},
\end{eqnarray*}

where CD is the background-corrected CD signal in mdeg, *C* is the DNA concentration in mol/l, and *L* is the path length of 1 cm.

Additionally, G4DNA thermal stability in CsCl buffer as well as upon hemin addition in KCl buffer was tested using CD melting of 2 µM G4DNA. The CD melting was performed using the AppliedPhotophysics qCD spectrometer using 2.5 ml samples with a DNA concentration of 2 µM in a clear, 10-mm, 3-mL quartz cuvette from 9°C to 94°C at a ramp of 0.5°C/min with magnetic stirring. Data analysis was performed using the Global3 analysis software (Global 3 Thermal Global Analysis Software) using a two-state model with no baseline correction.

### Fluorescence spectroscopy

The fluorescence from TOTO™-1 and SYTO™60 was measured at room temperature using the fluorimeter, FluoroMax-3 by HORIBA Jobin Yvon Inc. using a 3 × 3-mm, 60 µl quartz cuvette (Hellma™). Excitation wavelengths of 480 and 630 nm were used to excite TOTO™-1 and SYTO™60, respectively. Emission was measured from 490–800 nm and 640–800 nm. Excitation and emission bandpass slits of 5 nm were used for all experiments. An integration time of 0.5 s was used, and a step increment of 1 nm.

Additionally, fluorescence measurements were conducted for *K*_D_ determination using the plate reader, CLARIOstar Plus by BMG LABTECH, using black 100-µl, flat bottom 384-well plates (BRAND, 781622). Excitation wavelengths of 500 and 635 nm were used to excite TOTO™-1 and SYTO™60, respectively, and emission was measured from 514–599 nm and 650–720 nm. Excitation and emission bandpass slits of 5 nm were used for all experiments. An integration time of 0.1 s with 20 excitation flashes per well was used, and a step increment of 3 nm.

### Absorption spectroscopy

Absorption measurements from SYTO™60 and TOTO™-1 free in solution and upon binding to G4DNA or dsDNA were performed using the UV-Vis Spectrometer, Cary 60 by Agilent Technologies, using a 10-mm, 50-µl quartz cuvette (Hellma™). Measurements were recorded at room temperature, and spectra were acquired between 200 and 800 nm with a scanning speed of 60 nm/min. A spectrum from the buffer solution alone (100 mM KCl and 20 mM Tris, pH 7.5) was recorded and subtracted as background from all following measurements. SYTO™60 and TOTO™-1 were measured at 2 µM in buffer, either with or without 5 µM DNA (G4DNA or dsDNA) added.

### Characterization of DNA-binding dyes: *K*_D_ determination using synthetic DNA

Oligonucleotides were thermally annealed in the assay buffer (100 mM KCl or CsCl and 20 mM Tris–HCl, pH 7.5) at 35.8 µM by heating the samples to 95°C for 5 min in a water bath, followed by slow cooling overnight. The dissociation constant (*K*_D_) was determined from a titration series with DNA concentrations from 20.4 µM to 20 nM, made using a dilution series in buffer on the day of the experiment. TOTO™-1 or SYTO™60 was added to a final concentration of 0.5 µM just before measurement. Samples were measured from lowest to highest DNA concentration, with the same time interval of around one minute from the addition of the dye to the measurement of the sample. The kinetics were observed to be very fast, even at low concentrations, leading to equilibrium conditions in under a minute.

The *K*_D_ was determined using the following binding model [30]:


\begin{eqnarray*}
\mathrm{Fraction}\,\,\mathrm{Bound} = \,\,\frac{{a \cdot \left[ {\mathrm{ DNA}} \right]}}{{{K_\mathrm{ D}} + \left[ {\mathrm{ DNA}} \right]}},
\end{eqnarray*}

where the Fraction Bound is the background-corrected integrated emission from TOTO™-1 (514–599 nm) or SYTO™60 (650–719 nm), [DNA] is the DNA concentration, and *a* is a scaling factor. The standard deviation was determined from the mean of at least three individual repeats. The number of repeats is reported in Supplementary Table S1. Statistical significance was found using a symmetrical, two-tailored Student’s *t*-test with variances tested using the *F*-test, and, if different, *t*-values were found using Welch’s *t*-test.

### A/D ratio determination using synthetic DNA

Oligonucleotides were thermally annealed in the assay buffer (100 mM KCl or CsCl and 20 mM Tris–HCl, pH 7.5) at 17.5 µM by heating the samples to 95°C for 5 min in a water bath, followed by slow cooling overnight. The DNA was diluted in buffer to a concentration of 0.5 µM for all experiments, while one of the dye concentrations was kept constant at 0.5 µM and the other was either 0, 0.5, 1, 2, or 3 µM. For each sample condition, two spectra were recorded: one where TOTO™-1 was excited, and the emission from both dyes was measured (FRET spectra), and one where SYTO™60 was excited, and the direct emission from SYTO™60 was measured. The acceptor/donor (A/D) ratio was calculated as follows:


\begin{eqnarray*}
\mathrm{ A/D}\,\,\mathrm{ratio} = \,\,\frac{{{I_{\mathrm{FRET}}}}}{{{I_{\mathrm{TOTO} - 1}}}},
\end{eqnarray*}

where *I*_FRET_ is the peak intensity at 685 nm from SYTO™60 upon TOTO™-1 excitation, and *I*_TOTO-1_ is the peak intensity at 545 nm from TOTO™-1 upon TOTO™-1 excitation. All spectra were background-corrected before analysis. Results are from minimum of three independent measurements (3, 6, or 8 measurements), and the A/D ratio error was determined as the standard deviation of the mean.

### FRET experiments with synthetic DNA and DNase I

Oligonucleotides were thermally annealed as described in the previous section. The DNA was diluted to 0.5 µM in buffer (6× reaction buffer and 100 mM KCl) for all experiments and split into two batches. The first batch was treated with DNase I (New England Biolabs, 0–114 units/ml) for 1 h at room temperature, while the second batch was untreated. TOTO™-1 and SYTO™60 were added thereafter, and fluorescence spectra were recorded. Experiments were repeated three times, and the standard deviation of the mean was plotted as a shaded area around the fluorescence spectra.

### Urea-PAGE of synthetic DNA with DNase I

The DNase I degradation of oligonucleotides was further analyzed using denaturing urea polyacrylamide gel electrophoresis (Urea-PAGE). Urea gel (12%, 20 × 20 × 0.3 cm^3^) was made from monomeric UreaGel concentrate (29:1) and UreaGel diluent (National diagnostics), 10×Tris/borate/ethylenediaminetetraacetic acid (TBE), 10% ammonium persulfate and tetramethylethylenediamine according to supplier’s instructions and polymerized for 1 h. The gel was set to pre-run for 30 min in 1× TBE at constant voltage (556 V, ∼28 W).

Oligonucleotides were thermally annealed as in the previous section. On the day of the experiment, thermally annealed oligonucleotides were diluted (G4DNA: 3.37 µM, ssDNA: 3.37 µM, dsDNA: 0.56 µM) in buffer (6× reaction buffer, 100 mM KCl) with DNase I (0–100 units/ml) and incubated at room temperature for 30 min. The reaction was terminated by the addition of loading buffer (final concentrations of 51% formamide, 7.2% glycerol, 0.09% sodium dodecyl sulfate, and 9.0% EDTA), diluting the DNA samples to 1.35 µM for G4DNA and ssDNA, and 0.22 µM for dsDNA. The samples were denatured by heating to 95°C for 10 min just before they were loaded on the gel. All wells were cleaned with 1× TBE to remove excess urea, and each well was loaded with a 500 ng DNA sample. The Ultra Low Range DNA Ladder (Thermo Fisher) was used as a size marker. Electrophoresis was performed for 30 min with a metal plate attached to distribute the heat evenly. It was left to cool down for 10 min and then stained in 1× TBE with 1× SYBR™Gold for 5 min, followed by detaining in 1× TBE for 5 min. The gel was scanned using the Typhoon FLA scanner (Amersham Typhoon, Gel and Blot Imaging System) from GE Healthcare, using the SYBR™Gold setting (excitation at 488 nm, long-pass filter at 550 nm, pixel size of 100 µm). Gel scans were analyzed using the Fiji (ImageJ) software [31]. The percentage of non-degraded DNA was quantified as the percentage of “higher bands” divided by the sum of “higher and lower bands” for each lane profile.

### Microbial cultures and growth conditions

Autoclaved BHI (Merck, 53286) 37 g/l was used for agar plates (15 g/l agar in BHI) as well as overnight cultures (16–20 h at 37°C with 180 rpm shaking). Autoclaved TSB (Merck, T8907) 30 g/l supplemented with 100 mM NaCl (TSB-NaCl), 100 mM KCl (TSB-KCl), or 100 mM CsCl (TSB-CsCl) was used for biofilm growth. In some experiments, the media was also supplemented with 5 µM hemin (denoted as e.g. TSB-NaCl-hemin).

The clinical isolates of *S. epidermidis* AUH4567 [32], *S. epidermidis* strain 1457 wildtype and 1457 ΔatlE mutant (kindly provided by Prof. Dr Holger Rohde, Universitatsklinikum Hamburg-Eppendorf, Hamburg, Germany), and *P. aeruginosa* (PAO1 DSM 19880) were streaked onto BHI agar. Overnight cultures were inoculated from single colonies, and biological replicates were inoculated from separate colonies. Biofilms were inoculated from overnight cultures diluted 20× into TSB in 96-well plates (#1.5 polymer coverslip, IbiTreat tissue culture-treated, sterile, square well, Ibidi #89626) and incubated at 37°C at 150 rpm for 6 h for initiation of biofilm formation. The media were then replaced with the designated media for the specific experiment and subsequently exchanged daily for 3 days (for *S. epidermidis* biofilms) or 10 days (for *P. aeruginosa* biofilms).

### Staining, microscopy, and image analysis

#### Confocal laser scanning microscopy

All biofilms were visualized by confocal laser scanning microscopy (CLSM) (Zeiss LSM700 or LSM900) using a 63× oil immersion objective (PlanApochromat, NA 1.4), and the laser/gain settings used are noted for each experiment below. All images shown are representative from biological replicates.

#### Visualization of bacterial cells

Bacteria were visualized by intracellular DNA staining using SYTO™60 (639 nm excitation, 640–750 nm emission), SYTO™41 (405 nm excitation, 410–480 nm emission), or the membrane-binding stain FM4-64 (488 nm excitation, 600–750 nm emission). Specific concentrations are given for each experiment in the description below.

#### Visualization of DNA by immunolabeling

The *S. epidermidis* AUH4567 biofilms were immunolabeled in the solutions of the antibodies BG4 (1:100) or 1H6 (1:100) in 3% BSA in 1× PBS (45 min) at room temperature. The 3% BSA in 1× Pierce™ PBS blocking buffer was used as a blocking agent prior to immunolabeling as well as the washing solution in steps prior and after immunolabeling. When G4 was immunolabeled together with B-DNA, immunolabeling was carried out in three steps: Step 1, where BG4 (1:100) was incubated for 45 min followed by a washing; Step 2, where BG4 and AB1 (both at 1:200) were incubated for 45 min followed by a washing; Step 3, for labeling of AB1 with the secondary antibody AB2 (1:150) for 45 min followed by washing. The fluorescence of CF405S-conjugated AB1–AB2 was excited using a 405 nm laser. The fluorescence of Atto-488-conjugated BG4 and 1H6, as well as the FM 4-64, was excited using a 488 nm laser, and G4s were detected in ch1 using a short-pass 630 nm filter (Atto488 emission), while cellular membranes were detected in ch2 using a long-pass 630 nm filter (FM 4-64 emission).

#### Visualization of DNA by FRET

For visualizing G4 in biofilms using FRET, biofilms were stained by 2.5 µM TOTO™-1 and 12.5 µM SYTO™60 in 100 mM NaCl (10 min). The TOTO™-1 fluorophore was excited using a 488 nm laser, and fluorescence was imaged in two channels: in ch1 using a short-pass 630 nm filter [TOTO™-1 donor emission following donor excitation (DD)], and in ch2 using a long-pass 630 nm filter [FRET signal from SYTO™60 acceptor emission following donor excitation (DA)]. The SYTO™60 fluorophore was excited by a 639 nm laser, and fluorescence was imaged in ch2 using a long-pass 630 nm filter [acceptor emission of SYTO™60 following direct acceptor excitation (AA)]. The detected fluorescence was assigned the following false coloring: green (TOTO™-1, DD, dsDNA), red (FRET, DA, G4 DNA), and blue (SYTO™60, AA, intracellular DNA). In experiments in *P. aeruginosa biofilms*, the filter’s wavelength cutoff was 590 nm instead of 630 nm.

#### Image analysis

The CLSM images were analyzed using a custom-made script (ERDA Digital Repository, https://github.com/LineLund95/FRET-ratio-fiji-macro) that determined the pixel A/D ratio and pixel A-D difference (A/D_p_ ratio and A-D_p_ difference). The A/D_p_ ratio is the background-corrected red pixel intensity divided by the background corrected green pixel intensity. The background subtraction is done for the A/D_p_ ratio images to remove unwanted noise from dividing background intensities with each other, leading to unrealistic ratios and introducing possible bias. We determined the percentage of all green pixels with an A/D_p_ ratio above 1 [A/D_p_ ratio > 1 (%)]. The A-D_p_ difference is the red pixel intensity minus the green pixel intensity before background correction. Here, the pixel intensity range is set from 0 to 15 in all images. We used a background intensity of 5 for all images and an intensity saturation of 10% of the pixels for enhanced contrast of green/red images for visualization, unless otherwise specified. The standard deviation was determined from the mean of 5 (TOTO™-1 only) and 8 images (TOTO™-1 and SYTO™60). Statistical significance was found using a symmetrical, two-tailored Student’s *t*-test with variances tested using the *F*-test, and if different, *t*-values were found using Welch’s *t*-test.

### Sample preparation and image acquisition settings for specific experiments

#### Experiment #1: Visualization of native and externally supplemented G4s in *S. epidermidis* biofilms by FRET

*Staphylococcus epidermidis* AUH4567 biofilms were grown in (i) TSB-NaCl (five biological replicates), (ii) TSB-NaCl with 5 µM 4×G4DNA (five biological replicates), or (iii) TSB-NaCl hemin (five biological replicates) and imaged using FRET. Some biofilms from (i) and (ii) were treated with DNase I (Roche) or MNase (Thermo Fisher Scientific), as stated above (three biological replicates), to assess visualization of G4s when the dsDNA was removed from the sample. Biofilms were labeled with SYTO™60 and TOTO™-1 for FRET imaging as described earlier. Image acquisition was performed using the following settings. Biofilm samples from (i) and (ii) used excitation at 488 nm (3.0% power), ch1 had a 800 gain, ch2 had a 750 gain, and excitation at 639 nm (1.5% power), ch2 had a 750 gain. Biofilm samples from (iii) were imaged using an excitation at 488 nm (4.0% power), ch1 had an 800 gain, and ch2 had 800 unless otherwise specified, and excitation at 639 nm (1.5% power), ch2 had a 750 gain. The image histograms were adjusted to 1–255 for the green channel (DD), 1–100 for the red channel (DA), and 100–255 for the blue channel (AA).

#### Experiment #2: Visualization of native and externally supplemented G4s in *S. epidermidis* biofilms by immunolabeling to compare with FRET-based G4 detection in Experiment 1

*Staphylococcus epidermidis* AUH4567 biofilms were grown in (i) TSB-NaCl (five biological replicates), (ii) TSB-NaCl with 5 µM 4×G4DNA (three biological replicates), or (iii) TSB-NaCl-hemin (five biological replicates) and imaged using fluorescence immunolabeling as described earlier. Bacterial cells were visualized by membrane staining using FM4-64 (10 mg/l, 30 min). For image acquisition, Atto488-conjugated BG4 was excited at 488 nm (4.5% power) and emissions were recorded at <600 nm with varied gain settings (900–1100). CF405S-conjugated AB2 antibody was excited at 405 nm (4.5% power) and emissions were recorded <550 nm with varied gain settings (1000–1100). FM4-64 was excited at 488 nm (2% power) and emissions were recorded at 630–800 nm using a gain of 800–900. The image histograms were adjusted to 1–255 for the green channel (AB2), 1–255 for the red channel (BG4), and 100–255 for the blue (FM4-64) channel.

#### Experiment #3: Visualization of native G4s in *S. epidermidis* biofilms by immunolabeling

To further validate the presence of native G4s in *S. epidermidis* biofilms grown in TSB-NaCl, we also visualized G4s using different G4-specific antibody and compared G4 detection in biofilms grown in conditions that promote G4 formation (TSB-NaCl and TSB-KCl), prevent G4 formation (TSB-CsCl), or using an eDNA-negative mutant strain that is unlikely to form extracellular G4s at all. *Staphylococcus epidermidis* biofilms were grown from 1457 wildtype and strain 1457 ΔatlE lacking eDNA production (one biological replicate of each) in the TSB-NaCl, TSB-KCl, or TSB-CsCl. G4s were visualized by immunolabeling using antibody 1H6. Total eDNA was visualized by TOTO™-3 staining (2.5 µM), and bacterial cells were visualized by intracellular DNA labeling with SYTO™41 (12.5 µM). For image acquisition, SYTO™41 was excited at 405 nm (3.0% power) and emissions were recorded at 300–550 nm (Gain 750), Atto488-conjugated 1H6 was excited at 488 nm (4.5% power) and emissions were recorded at 509–800 nm (Gain 900). TOTO™-3 was excited at 639 nm (4.0% power) and emissions were recorded at 644–800 nm (Gain 950). The image histograms were adjusted to 1–70 for the green channel (TOTO™-3), 1–70 for the red channel (1H6), and 1–140 for the blue channel (SYTO™41).

#### Experiment #4: Visualization of G4s by FRET in *P. aeruginosa* biofilms to demonstrate the use of this method in another species

*Pseudomonas aeruginosa* PAO1 biofilms (two biological replicates) were grown in the TSB-NaCl and labeled with SYTO™60 and TOTO™-1 for visualization of G4 by FRET as described earlier. Image acquisition settings: lasers 488 nm (0.2% power, ch1 750 gain, ch2 750 gain) and 639 nm (0.2% power, ch2 750 gain). The image histograms were adjusted to 20–255 for the green channel (DD), 20–60 for the red channel (DA), and 100–255 for the blue channel (AA).

#### Experiment #5: Visualization of G4s in *S. epidermidis* biofilms by immunolabeling and NMP staining to assess NMP’s suitability for detection of extracellular G4s in biofilms

*Staphylococcus epidermidis* 1457 biofilms (two biological replicates) were grown in the TSB-NaCl, and G4s were visualized by immunolabeling with antibody BG4 (see earlier) and NMP staining (20 µM, 30 min). In biological replicate 1, the extracellular B-DNA was visualized by TOTO™-1 staining (2.5 µM, 30 min). In biological replicate 2, the extracellular B-DNA was visualized by immunolabeling using antibodies AB1–AB2. Biological replicate 1: BG4 was excited at 488 nm (4.0% power) and emission was recorded at 300–590 nm (gain 950). The AB2 and NMP were excited at 405 nm laser (4.0% power), and emissions were simultaneously recorded as two channels at 300–590 nm (gain 950) for AB2 and 590–800 nm (gain 1100) for NMP. Biological replicate 2: BG4 was excited at 488 nm (4.0% power) and emissions were recorded at 300–590 nm (Gain 950). NMP was excited at 405 nm (4.0% power) and emissions were recorded at 590–750 (Gain 950). TOTO™-1 was excited at 488 nm (2.0% power) and emissions were recorded at 509–800 nm (Gain 700). The image histograms were adjusted to 1–255 for the green channel (AB2 or TOTO™-1), to 1–255 for the red channel (BG4), and 100–255 for the blue channel (NMP).

## Results

### SYTO™60 and TOTO™-1 display different DNA binding affinities

**Figure 1.**
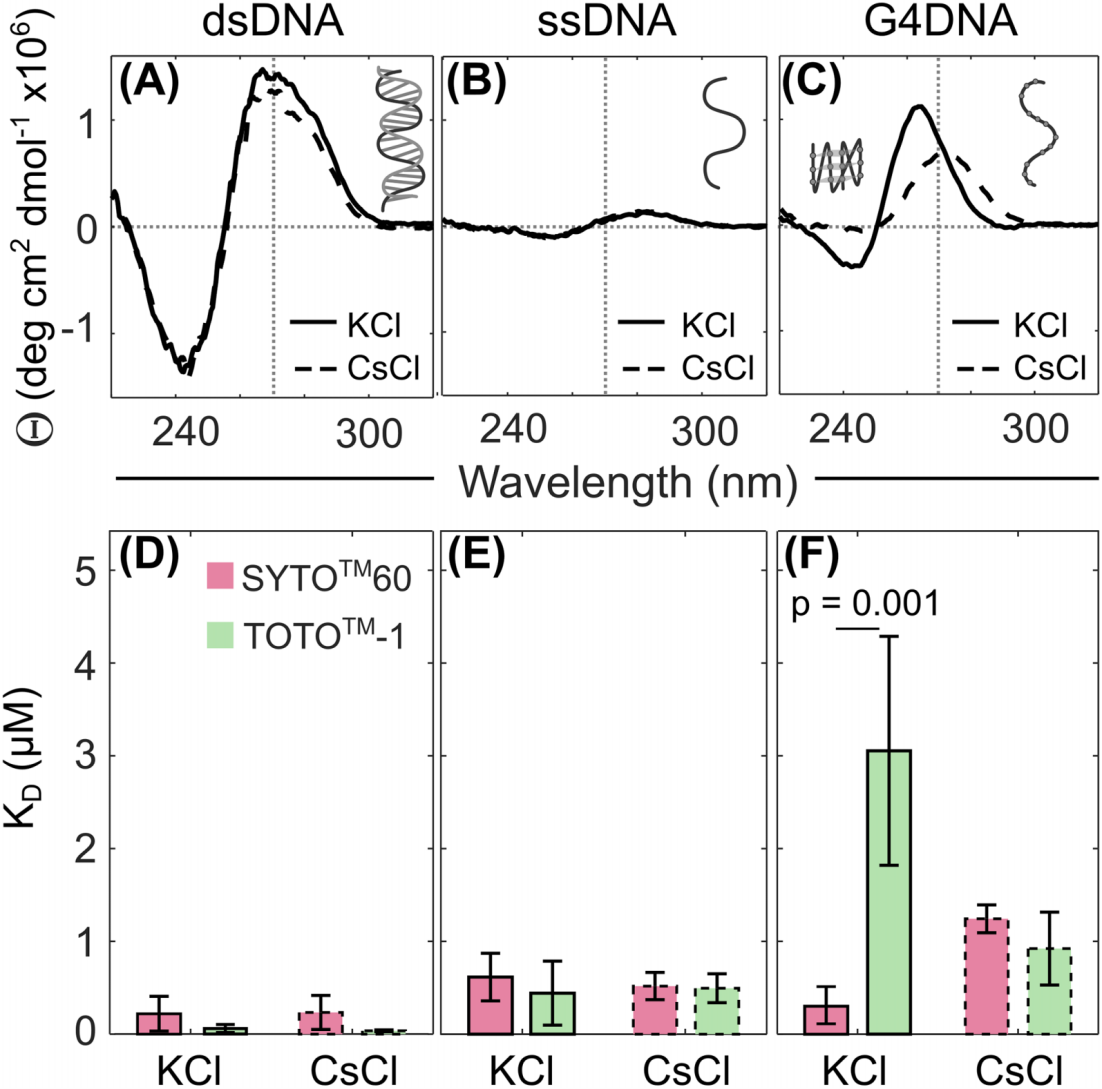
Preferential binding affinities of SYTO™60 and TOTO™-1 to DNA structures. CD spectra for (**A**) dsDNA, (**B**) ssDNA, and (**C**) G4DNA in either KCl-containing (full line) or CsCl-containing (dashed line) solutions. Gray horizontal and vertical lines indicate a CD signal of zero and a CD signal at 270 nm, respectively. Illustrations of the DNA constructs are shown in each plot. Below are dissociation constants, *K*_D_, for SYTO™60 (pink) and TOTO™-1 (green) binding to (**D**) dsDNA, (**E**) ssDNA, and (**F**) G4DNA. The *P*-value below .01 from *t*-tests between different samples is indicated. (See Supplementary Fig. S2 for fluorescence binding curves and Supplementary Table S1 for *K*_D_ values).

To evaluate the suitability of the DNA-binding dyes TOTO™-1 and SYTO™60 for G4 detection, we first investigated their binding affinities to double-stranded, single-stranded, and G4 DNA using constructs dsDNA, ssDNA, and G4DNA from Table 1. Model constructs for single- and dsDNA were chosen with high G content (44% in ssDNA) to account for a preference of TOTO™-1 and SYTO™60 for G-bases that could result in a false-positive FRET signal that is not linked to G4 detection. Both TOTO™-1 and SYTO™60 showed almost no fluorescence signal in the absence of DNA and at least an order of magnitude increase in fluorescence signal in the presence of DNA (Supplementary Fig. S1). The excitation wavelengths were kept constant between the different DNA samples and were chosen close to the dyes’ absorption peaks (Supplementary Fig. S1). To test the dyes’ sensitivity to folded G4s, samples were prepared in the presence of either 100 mM K^+^ or 100 mM Cs^+^, conditions that favor or inhibit G4 folding, respectively. We validated the secondary structure for the different DNA sequences using CD spectroscopy (Fig. 1A–C) before proceeding to measure the *K*_D_ of DNA-TOTO™-1 and DNA-SYTO™60 complexes by varying the DNA concentration while keeping the dye concentration constant (Fig. 1D–F, Supplementary Fig. S2, and Supplementary Table S1).

The dsDNA sample showed a pronounced increase in the CD signal around 240 and 270 nm, in both potassium- and cesium-rich buffer conditions, indicating helical duplex formation [33] (Fig. 1A). Additionally, the buffer conditions did not influence the measured binding affinity between dsDNA and TOTO™-1 or SYTO™60 (Fig. 1D). We note that TOTO™-1 binding to the dsDNA duplex had a very low *K*_D_ (below 100 nM, Supplementary Table S1). For ssDNA, the low CD signal indicated mostly unstructured DNA (Fig. 1B), and SYTO™60 and TOTO™-1 had comparable binding affinities in both buffers (Fig. 1E). The binding affinity to ssDNA was especially lower for TOTO™1 compared to the dsDNA, most likely since it is unable to bind via the bis-intercalation mechanism spanning the minor groove of duplex DNA. For G4DNA, we observed differences between G4 formation in KCl and CsCl buffer when inspecting the structural signatures using CD spectroscopy (Fig. 1C). In KCl, a positive peak around 260 nm is consistent with G4 formation with parallel topology, whereas the sample in CsCl (which inhibits G4 formation) showed a less intense peak around 270 nm, indicating a more helical, unstructured topology [34]. The intensity of this peak in CsCl increased upon slow annealing, suggesting the formation of aggregates at room temperature, which was further supported by the appearance of higher bands on a native gel (Supplementary Fig. S3). However, these higher bands contributed <8% to the total lane intensity, and aggregation could therefore be neglected. We observed different *K*_D_ values depending on G4 formation (Fig. 1F). In KCl, G4DNA preferred binding SYTO™60 (*K*_D_ below 1 µM) over TOTO™-1, whereas in cesium-containing buffer, G4DNA bound SYTO™60 and TOTO™-1 without preference.

From these results, we conclude that the folded G4 structure (G4DNA oligo) prefers binding SYTO™60 (*K*_D_ < 1 µM) over TOTO™-1 (*K*_D_ > 1 µM) in G4-forming conditions (KCl), whereas dsDNA prefers to bind TOTO™-1 (*K*_D_ << 1 µM) over SYTO™60 (*K*_D_ < 1 µM). ssDNA has similar affinity toward SYTO™60 and TOTO™-1 (*K*_D_ ∼ 1 µM). Thus, we conclude that in the presence of both dyes, the G4 will favor the binding of SYTO™60.

### G4 DNA motifs promote energy transfer between TOTO™-1 and SYTO™60

**Figure 2.**
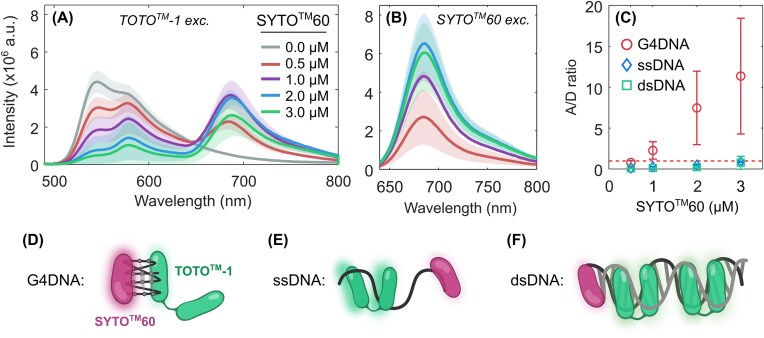
G4-specific red shift in TOTO™-1 fluorescence spectrum is due to energy transfer. Background-corrected emission spectra upon (**A**) TOTO™-1 and (**B**) SYTO™60 excitation with G4DNA. The samples are all measured at constant DNA (0.5 µM) and TOTO™-1 concentrations (0.5 µM) and varying SYTO™60 concentrations (0–3 µM) in potassium-containing buffer. (**C**) A/D ratio plots for G4DNA (red circles), ssDNA (blue diamonds), and dsDNA (green squares). The dotted line marks an A/D ratio = 1. (*N* ≥ 3). Below, illustrations of SYTO™60 and TOTO™-1 bound to (**D**) G4DNA, (**E**) ssDNA, and (**F**) dsDNA are shown.

As the spectral characteristics of TOTO™-1 and SYTO™60 allow FRET, we compared the FRET signal strength for G4DNA, ssDNA, and dsDNA (Fig. 2 and Supplementary Figs S4–S6). We first kept the DNA concentration at 0.5 µM, TOTO™-1 concentration at 0.5 µM, and varied the SYTO™60 concentration from 0.0 to 3.0 µM in potassium-containing reaction buffer (Fig. 2). We observed binding of both dyes, resulting in a FRET peak around 680 nm (at the location of SYTO™60 emission) while excited at 480 nm. The FRET phenomenon was strongest for G-rich G4DNA (Fig. 2A and B), with a more pronounced increase in the A/D ratio at a higher SYTO/TOTO ratio (Fig. 2C). For ssDNA and dsDNA, the A/D ratio stayed below 1 for all SYTO™60 concentrations (Supplementary Fig. S4). An illustration of the expected binding of the two dyes to a G4, ssDNA, and dsDNA is shown in Fig. 2D–F.

The spectral signatures became broader upon increased TOTO™-1 concentration up to 3 µM while keeping a constant SYTO™60 concentration of 0.5 µM, leading to nonoptimal conditions for observing G4-related A/D ratios (Supplementary Fig. S5). It was previously shown that several dye molecules can bind the same DNA strand when staining DNA with TOTO™-1, which influences the fluorescence intensity, even resulting in an abrupt quenching of the fluorescence around a dye-base pair (bp) ratio of one [35]. This quenching was also observed here, which led to more complex spectral changes, most likely due to several close binding events. Moreover, the TOTO™-1 spectral shape at high dye concentrations was different depending on the DNA structures. G-rich ssDNA may also form intermolecular π–π stacking with TOTO™-1, resulting in red-shifted fluorescence [36].

A FRET signal was also detected from unfolded G4DNA in cesium-containing reaction buffer at higher SYTO™60 concentrations (Supplementary Fig. S6). The observed A/D ratio was lower for unfolded compared to folded G4 in potassium-containing reaction buffer for the same DNA sequence (Fig. 2C and Supplementary Fig. S6C). Thus, the FRET signal is much stronger for G4 structures. From these results, we present an approach to quantify the FRET effect as an A/D ratio, where the FRET signal has a preferential sensitivity to G4s at SYTO™60/TOTO™-1 ratios > 1.

### Cleaving B-DNA by DNase I improves G-quadruplex DNA detection in complex solutions

**Figure 3.**
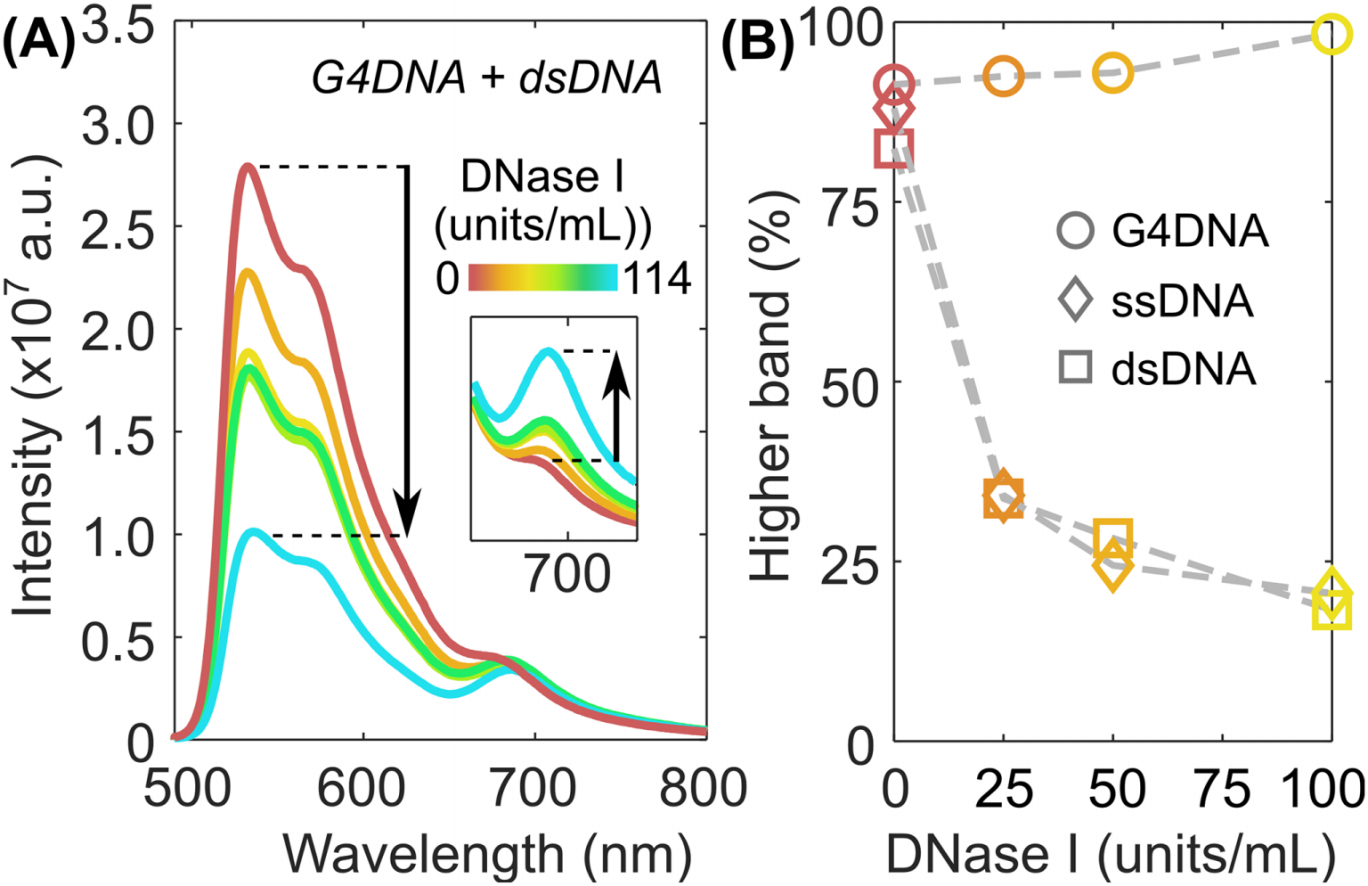
G4 DNA is resistant to DNase I treatment while single- and dsDNA are degraded. (**A**) Fluorescence spectra at equimolar concentration of G4DNA and dsDNA (0.5 µM) with TOTO™-1 and SYTO™60 added, showing the fluorescence intensity upon TOTO™-1 excitation with 0, 14, 29, 57, 86, and 114 units/ml DNase I. The zoom-in shows the relative increase in the FRET peak when all spectra have been normalized to the total area. (**B**) DNA degradation after DNase I treatment using denaturing Urea-PAGE with 500 ng of DNA in each well for G4DNA (round), ssDNA (diamond), and dsDNA (square). The quantification of the higher (non-degraded) bands is shown for each sample condition. See Supplementary Fig. S7 for the full Urea-PAGE image and lane profiles used in the analysis.

We noticed that ssDNA and dsDNA showed high TOTO™-1 fluorescence compared to G4DNA samples (Fig. 2A and Supplementary Figs S1A and S4A, D), and wondered if G4s can be detected in a complex sample containing different DNA structures. We prepared an equimolar mixture of G4DNA and dsDNA and observed that the spectral response in this cuvette experiment with homogeneous high dsDNA concentration was dominated by dsDNA (Fig. 3A and Supplementary Fig. S4). One way to deal with this problem is to remove dsDNA. An endonuclease, DNase I, was hereby used to degrade single- and dsDNA while leaving G4 structures untouched [37, 38]. As expected, the TOTO™-1 fluorescence intensity decreased with increased DNase I concentration (Fig. 3A), the TOTO™-1 fluorescence spectra changed shape with the two peaks becoming closer in intensity, and fluorescence from SYTO™60 was excited via energy transfer (Fig. 3A and its figure inset). These features are characteristic of G4 binding, as seen in Fig. 2A, and became more pronounced at higher DNase I concentrations where dsDNA gets degraded while G4DNA remains intact. The effect of DNase I was further confirmed using a denaturing Urea-PAGE. The three DNA samples were treated with an increasing amount of DNase I (0–100 units/ml) and run on a gel to assess DNA degradation. The G4DNA sample showed no degradation by DNase I, while both the dsDNA and ssDNA were almost completely broken down by the enzyme (Supplementary Fig. S7). A quantification of this degradation showed that the original G4 band stayed above 90%, while both ssDNA and dsDNA dropped below 35% in the presence of >25 units/ml of DNase I (Fig. 3B).

Thus, G4s could not be detected in a homogeneously mixed sample with high concentrations of dsDNA because the strong fluorescence from TOTO™-1 bound to dsDNA had “hidden” the FRET signal. However, this can be overcome by enzymatically degrading dsDNA, indicating that such enzyme treatment can enhance the detection of G4s by FRET in complex samples.

### G4 DNA is detected in *S. epidermidis* biofilm using FRET

In the experiments earlier, we detected G4s in a complex but well-defined sample of DNA oligos with high local concentration of dsDNA. Our next goal was to establish an approach that could be used to detect G4s among eDNA from complex biological samples, such as bacterial biofilms. Here, we validate our FRET approach using *S. epidermidis* as it forms biofilms that are rich in eDNA [39]. We additionally show that a FRET signal was also detected using *P. aergunosa* biofilms, where G4s were initially reported [6].

**Figure 4.**
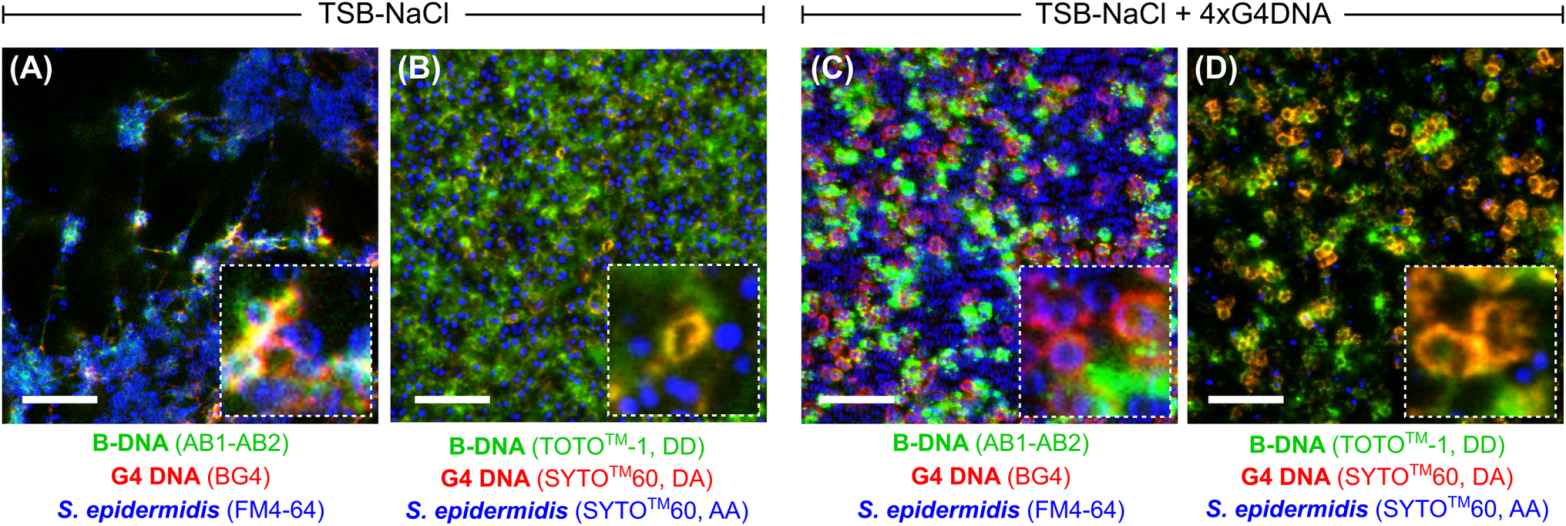
A G4-enriched FRET signal is found in the extracellular matrix of *S. epidermidis* (AUH4567) upon simultaneous binding of SYTO™60 and TOTO™-1, and the FRET localization is found in agreement with immunolabeling. (**A, C**) Immunolabeling of the biofilms with antibodies BG4 (red), AB1–AB2 (green), and cellular dye FM 4-64 (blue) following procedures in Experiment #2 in “Materials and methods” section. (**B, D**) TOTO™-1/SYTO™60 staining of the biofilms with TOTO™-1 excitation and emission detection (em. < 630 nm) in green and emission in the FRET spectral region (em. > 630 nm) in red, and direct excitation of SYTO™60 and detection of its fluorescence (em. > 630 nm) in blue following procedures in Experiment #1 in “Materials and methods” section. Biofilms were obtained in panels (A, B) TSB media with 100 mM NaCl, or (C, D) TSB-NaCl with 5 µM 4×G4DNA added to the media. Scale bar is 10 µm, and zoom-in images are 5 × 5 µm^2^.

*Staphylococcus epidermidis* only has a GC content of around 35%, and we could not predict abundant G4s based on its DNA sequence [40]. However, we previously established an *S. epidermidis* biofilm model that contained G4 and Z-DNA structures in the extracellular matrix by growing the biofilm in TSB media with 100 mM NaCl and 5 µM hemin with incubation at 150 rpm agitation [7]. In this model, G4 DNA was detected via immunofluorescence labeling both in the absence and presence of hemin, while Z-DNA was only detected in the presence of hemin. We used the same model here in three variations where we expect formation of G4 DNA: (i) with NaCl (ii) with NaCl and 5 µM oligos pre-folded into G4 DNA (4×G4DNA), and (iii) with NaCl and 5 µM hemin. First, we confirmed the presence of the native and added G4s in the biofilms of *S. epidermidis* by immunolabeling with the antibodies BG4 and 1H6 (Fig. 4 and Supplementary Figs S8 and S9). We noticed a reproducible formation of web-like eDNA for G4 DNA detected via immunolabeling and the appearance of G4 DNA around bacteria cell surfaces, consistant with our previous report [7]. G4 DNA were predominantly associated with cell surfaces in G4-supplemented biofilms.

For the first time, we investigated biofilms using two-channeled fluorescence upon excitation of TOTO™-1: below 630 nm (green) showing emission from TOTO™-1, as well as above 630 nm (red) showing emission from SYTO™60 via energy transfer from TOTO™-1. In a second track, we used 639 nm excitation to detect intrinsic fluorescence from SYTO™60 above 630 nm upon excitation of SYTO™60 (blue). We observed a signal in the FRET channel in all three variations of the biofilm model (Fig. 4 and Supplementary Fig. S10) both in the absence and presence of exogeneous G4 DNA or hemin. Furthermore, FRET imaging and immunolabeling visualized similar structural features in the biofilm with G4s-enriched signals located within the web-like eDNA and surface associated eDNA (Fig. 4 and Supplementary Fig. 10).

FRET images of a model biofilm using *P. aergunosa*, which has higher GC content (*∼*66.6% [41]) and for which there is report of G4s in the biofilm [6], showed a FRET signal with similar patterns as previously reported with immonolabeling (Supplementary Fig. S11). Taken together, our data are consistent with the FRET signal from TOTO™-1 and SYTO™60 locating to G4 DNA in the biofilms.

When using direct fluorescence from an established G4 fluorescence reporter for G4 imaging, only weak colocalization with eDNA and less clear spatial features were observed (Supplementary Fig. S12). A difficulty of this approach is that the G4 fluorescent dye goes inside the live bacteria cells, resulting in bright signals not associated with extracellular G4 DNA. The FRET approach detects primarily motifs associated with eDNA, as the donor TOTO™-1 is impermeable and mostly stains eDNA. This advantage of the FRET approach reveals G4 features within the eDNA and shows clearly that both web-like and cell-surface G4 DNA-enriched patterns are associated with eDNA, as the feature appear in both the green and red channels (Supplementary Fig. S10).

### Analysis of the FRET signal in *S. epidermidis* biofilms

**Figure 5.**
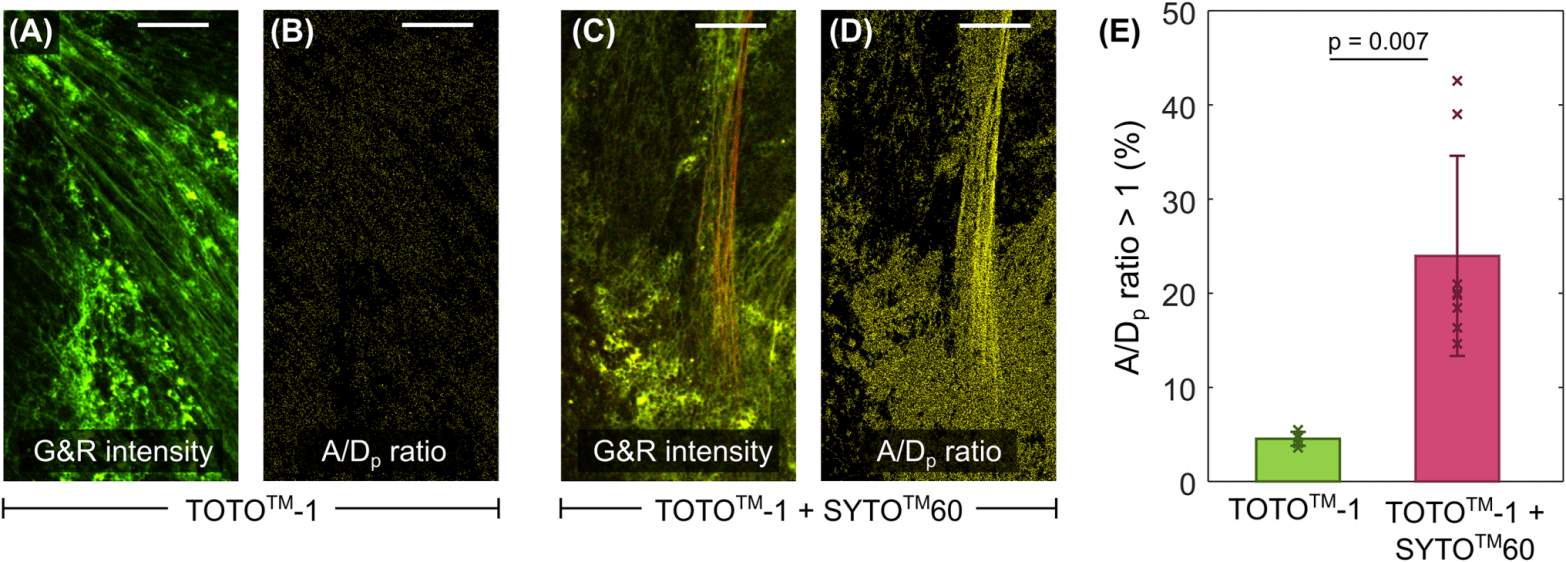
FRET is concentrated in the eDNA strings of *S. epidermidis* AUH4567 biofilms. (**A, B**) Biofilms produced in TSB-NaCl with 5 µM hemin stained with 2.5 µM TOTO™-1 alone and (**C, D**) 2.5 µM TOTO™-1 with 12.5 µM SYTO™60 and visualized using channel 1 (<630 nm, green) and channel 2 (>630 nm, red) following procedures in Experiment #1 in “Materials and methods” section. The green/red fluorescence is shown in the left panels (A, C), while pixels with an A/D_p_ ratio above 1 are shown in the right panels (B, D). A background value of 5 was used for all images. See Supplementary Fig. S13 for other background values. (**E**) The percentage of green pixels with a A/D_p_ ratio above 1. The *P*-value from *t*-test is indicated. Scale bar is 15 µm.

Visualization of G4 DNA in complex samples can be used to localize these motifs and can, in combination with quantitative analyses, be used to identify conditions that promote G4 formation or to identify enzymes or other therapeutics that disrupt G4 structures *in situ*. To establish the sensitivity of the FRET approach, we analyzed FRET images taken in the presence of hemin, as these conditions gave the lowest FRET signal (Fig. 5 and Supplementary Figs S10 and S13A, B). The presence of hemin appears to both increase the stability of G4s and influence a little G4-staining as suggested by our data with pure DNA motifs (Supplementary Figs S14 and S15). An increase in G4 stability likely makes these motifs easier to detect, while a possible competition between hemin and SYTO™60 binding, which would depend on concentrations, decreases the detection sensitivity.

For the analysis, we used both the “A-D_p_ difference” (FRET signal minus TOTO™-1 signal) and “A/D_p_ ratio” (FRET signal divided by TOTO™-1 signal) images determined from multi-channel fluorescence microscopy images such as the ones shown in Fig. 4. The first step for data analysis was to determine the background pixel values. It was adjusted to remove low-intensity signals resulting in high A/D_p_ ratios values that are the result of noise and not linked to the presence of fluorophores. In our case, using a background value of 5 was effective in removing this unwanted noise from A/D_p_ ratios images. The used background pixel value does not influence “A-D_p_ difference” images, which are used as reference for setting background values that remove the noise but do not remove the signal (see Supplementary Fig. S13). After background removal, the resulting image can be used to identify how many pixels have an A/D ratio above a certain threshold. This analysis was done for a wide range of thresholds (Supplementary Fig. S16). A threshold of 1 was used, since this gave detection of G4 DNA signals in cuvette-based experiments (Fig. 2) and worked well with our microscopy setup. Other thresholds would also be possible without loss of information (Supplementary Fig. S16). The images were quantified by measuring the percentage of pixels with an A/D_p_ ratio above 1, compared to the number of green pixels. This removed variances in biofilm density from the image analysis and reveals the percentage occurrence of FRET signal in the analyzed image.

Figure 5 shows two *S. epidermidis* biofilms stained using the same procedure and settings, one was stained with TOTO™-1 alone, and these conditions showed no FRET signal and the other was stained with both TOTO™-1 and SYTO™60 and showed FRET. The A/D_p_ ratio values >1 are shown in yellow (Fig. 5B and D) (Supplementary Fig. S13A and B for the A-D_p_ difference image). The A/D_p_ ratio representing FRET was significantly higher in biofilms stained with both TOTO™-1 and SYTO™60 compared to biofilms stained only with TOTO™-1, leading to an average signal-to-noise ratio of 5.3 (Fig. 5E and Supplementary Fig. S16). From these results, we conclude that our FRET apporach between TOTO™-1 and SYTO™60 in a complex sample gives a readout with good signal-to-noise ratio for the presence of G4 DNA motifs.

**Figure 6.**
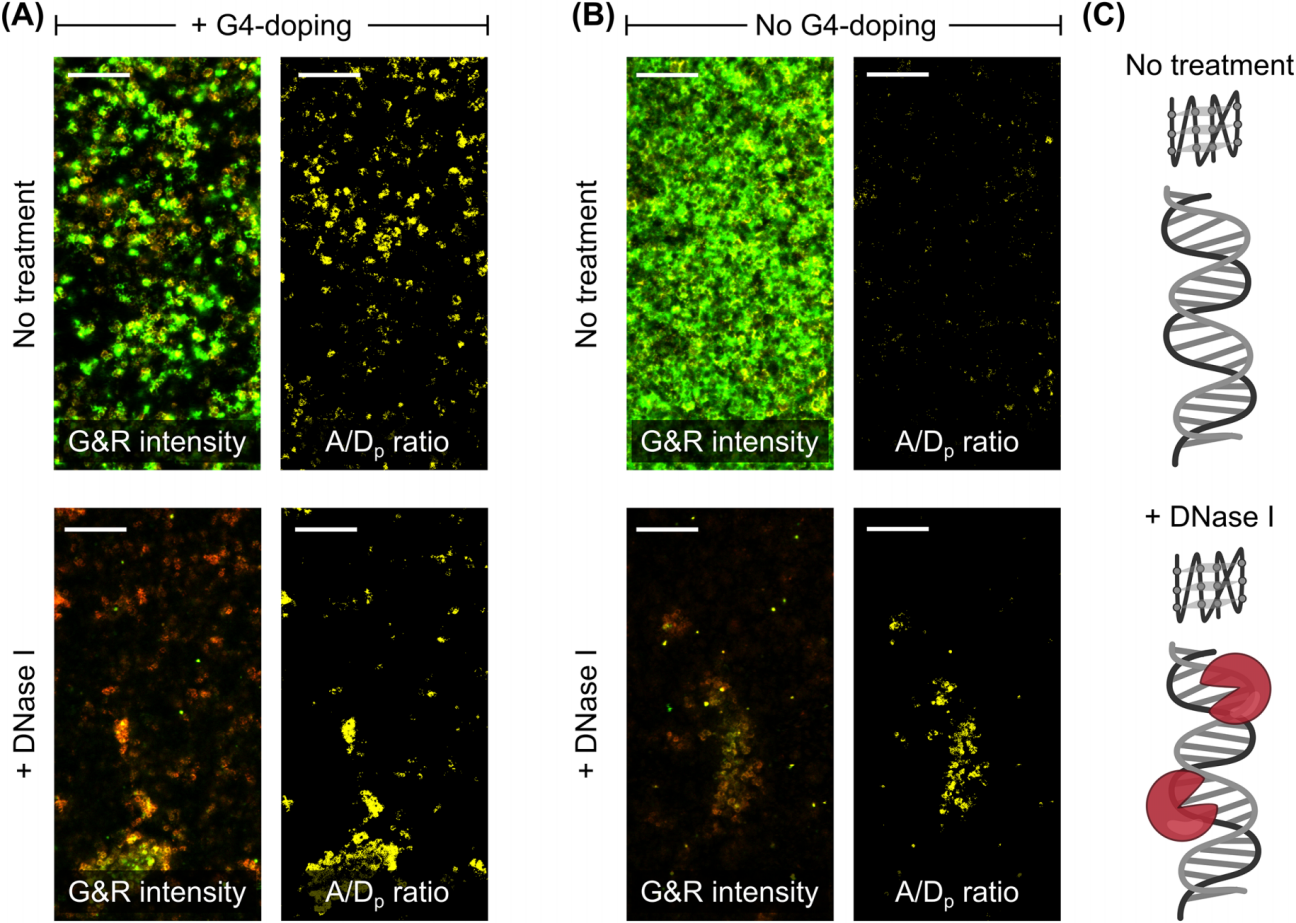
DNase I treatment uncovers additional FRET signal. Example images of 3-day *S. epidermidis* AUH4567 biofilms stained by 2.5 µM TOTO™-1 and 12.5 µM SYTO™60 with G4-supplement using the 4×G4DNA sequence (**A**) and without G4-supplement (**B**) following procedures in Experiment #1 in “Materials and methods” section. Images of *S. epidermidis* in TSB-NaCl using channel 1 (<630 nm, green) and channel 2 (>630 nm, red) with equally enhanced color brightness (G/R intensity) as well as the “A/D_p_ ratio” images are shown. Here, samples with no treatment (A and B, upper panels) and DNase I treatment (A and B, lower panels) are shown. Scale bar is 15 µm. (**C**) Illustrations of G4 DNA and dsDNA in biofilms with no treatment (upper illustration) and DNase I treatment (lower illustration).

The next step was to test if we could use the FRET signal to map G4 DNA motifs in biofilms treated with DNase I, as this enzyme boosted G4-mediated FRET in the pure DNA system by removing a significant portion of double- and ssDNA (Fig. 3). We additionally tested biofilm treatment with another enzyme, MNase, an endo-exonuclease that degrades both double- and single-stranded RNA and DNA with a preference for single-stranded regions and regions adjacent to G-stretches [37, 42]. We created two biofilm samples with low and high concentrations of G4 DNA in the extracellular matrix by simply adding 4×G4DNA to the growth media in the latter case. Subsequently, we treated biofilms with DNase I or MNase. Finally, we stained and visualized the resulting biofilms as described in Fig. 5, using an A/D_p_ ratio threshold of 1 (Fig. 6 and Supplementary Fig. S17).

The doped biofilms supplemented with exogenous G4 DNA generally showed high occurrence of FRET signals (A/D_p_ ratio above 1) throughout the image (Fig. 6, and Supplementary Fig. S17). The occurrence of the FRET signal remained high in the DNase I and MNase-treated biofilms, showing little enzymatic degradation of G4 DNA. We observed clusters in the FRET signal confirming that enzymatic treatment took place (Fig. 6 and Supplementary Fig. S17).

The plain undoped biofilms without 4×G4DNA supplement showed much lower occurrence of FRET, which increased upon DNase I and MNase treatment, indicating a higher ratio of G4 DNA compared to other DNA structures after enzyme treatment (Fig. 6B and C; Supplementary Fig. S17). Treatment with DNase I appeared to bring the larger increase in FRET signal occurrences and gives an approach to reveal the presence of possible G4 motifs not initially detected by FRET. Collectively, these results corroborate that the A/D_p_ ratio analysis can be combined with nuclease treatment for G4 imaging.

## Discussion

Most bacteria on Earth live in biofilms [43], and eDNA appears to be the one matrix component that these biofilms have in common [44]. For two decades, eDNA in biofilms was perceived as a structural component that together with polysaccharides affects biofilm elasticity, e.g. in the formation of streamers [45]. However, new research shows that eDNA contains secondary structures, such as Z-DNA and G4, that are resistant to mammalian DNase I [6, 7, 46, 47]. This discovery opens a new chapter in the understanding of how bacteria use eDNA for biofilm formation. Expanding our knowledge about G4 in environmental, industrial, and medical biofilms is hindered by the cumbersome and expensive immunolabeling techniques currently used to study these structures. A higher throughput method for detecting extracellular G4 in biofilms of different origins will be an asset for this novel and growing field.

In this study, we present new knowledge that is obtained using two well-known TO derivatives. Our novel approach uses energy transfer between the DNA-binding stains TOTO™-1 and SYTO™60 to detect and visualize G4 motifs by fluorescence microscopy. The method is >15× faster and 15× cheaper than immunolabeling. The cell-permeable red SYTO™60 fluorophore binds G4 DNA, ssDNA, and dsDNA with similar affinity (Fig. 1 and Supplementary Table S1), while the cell-impermeable green TOTO™-1 dye has a lower affinity for G4 DNA, although it binds both G4 DNA and dsDNA. We demonstrate that the binding of both dyes to G4 DNA results in a FRET signal that can be used to visualize G4s in the extracellular matrix of biofilms—a highly complex biological sample. This approach for G4 DNA imaging offers new possibilities for the detection of this non-canonical DNA structure in complex biological systems.

Our result has several implications. It reveals that the fluorescence intensity from TOTO™-1 staining of DNA is dependent on the DNA structure, and that G4 DNA might be overlooked when visualizing eDNA with standard imaging settings for TOTO™-1 and SYTO™60. This is partly due to TOTO™-1’s lower affinity to G4 DNA but is also a consequence of TOTO™-1 engaging as a donor in the FRET pair. A dim TOTO™-1 signal may therefore not reflect a lower eDNA concentration but rather a different eDNA conformation. On the other hand, the FRET signal provides a new opportunity to detect G4 DNA with simple fluorescent dyes rather than multi-step immunolabeling techniques. TOTO™-1 and SYTO™60 are routinely used for eDNA imaging in biofilms, and TOTO™-1 has been shown to be superior to common standards for live/dead staining [26]. The staining method proposed here is thus suited for live/dead staining, and for visualization of eDNA and G4 DNA in a single experiment, simply by adding a channel for detecting SYTO™60 fluorescence at TOTO™-1 excitation (FRET). SYTO™60 fluorescence (at SYTO™60 excitation) will visualize live cells, TOTO™-1 (at TOTO™-1 excitation) will visualize dead cells and eDNA, and the FRET signal (SYTO™60 fluorescence at TOTO™-1 excitation) will locate G4-enriched patterns in the eDNA. The method is thus suited for the analysis of G4 DNA in the extracellular matrix without interference from intracellular DNA and without need for an extra label.

We imaged the FRET signal in biofilms using confocal microscopy and quantified the area using the A/D_p_ difference and A/D_p_ ratio images. These two quantities give complementary information for assessing the presence of G4s. The A/D_p_ difference image contrast is strongly sensitive to fluorescence intensities, i.e. DNA abundance in the biofilm, while the A/D_p_ ratio is not, as it is a ratiometric value and, with adequate calibration, is a good indicator of the presence of G4s. We note that G-rich sequence effects alone also contribute to this FRET-based readout. Controls with synthetic oligos revealed indeed a FRET signal for highly G-rich oligos both in an unfolded (Supplementary Fig. S6) and folded conformation (Fig. 2). The folded configuration yielded a higher FRET signal, showing clear contributions from G4-structure effects to the FRET signal. We, however, cannot exclude that DNA sequence effects alone, which contribute to the FRET signal, may possibly give some false positive detection of G4s in the proposed FRET approach. To evaluate the extent of this limitation for G4 visualization in biofilms, we compared results obtained by our FRET approach to previously established immunolabeling approaches (Fig. 4 and Supplementary Figs S8 and S10). Both approaches gave very similar images in *S. epidermidis* biofilms, showing that the FRET method has a preferential sensitivity for G4 motifs.

Although *S. epidermidis* has a low genomic GC content, G4 DNA was clearly detected in *S. epidermidis* biofilm models in the presence of sodium and/or hemin. We generated a positive control sample by adding synthetic 4×G4DNA, which the bacteria incorporated into the biofilm. The location of G4 motifs around bacterial cells was confirmed by both immunolabeling and FRET (Fig. 4 and Supplementary Figs S8 and S10). In the absence of exogeneous G4 DNA, G4s appeared in different locations, including eDNA streamers that may also be responsible for the viscoelasticity of the biofilm, and around the surface of bacterial cells. We investigated how the eDNA composition responds to DNase I treatment and showed that the eDNA remaining after enzyme treatment had a strong FRET signal (Fig. 6), which indicates that G4 DNA in the eDNA matrix is DNase-resistant, which is consistent with previous reports [7, 47].

In conclusion, we provided a thorough biophysical characterization of FRET between TOTO™-1 and SYTO™60 in different DNA molecules and demonstrate its use for G4-enriched detection based on our observations from both pure DNA and eDNA in biofilm. We recommend collecting FRET data during standard eDNA imaging or live/dead staining assays with TOTO™-1 and SYTO™60, as this will indicate if G4 DNA structures are present before investigating further with complementary methods that are more expensive and time-consuming. This study paves the way for high-throughput analyses that can identify bacterial species, microbial communities, and natural or engineered environments that lead to the formation of G4s in bacterial biofilms, or the discovery of enzymes that can disrupt these resilient DNA structures.

## Supplementary Material

gkag712_Supplemental_File

## Data Availability

The data underlying this article are available through the Electronic Research Data Archive (ERDA) server at Aarhus University with the following link to the frozen archive: [https://www.erda.au.dk/archives/4763c9cc9fe0aef08011b1029dc96fe9/published-archive.html].
