# Supplementary material for: Detecting extracellular G4 DNA motifs in biofilms through energy transfer between DNA-binding dyes TOTO™-1 and SYTO™60
Source: Nucleic Acids Res. 2026 Jul 20;54(14):gkag712. doi: 10.1093/nar/gkag712 (PMC13384255; doi:10.1093/nar/gkag712)
Supplement: gkag712_Supplemental_File [file gkag712_supplemental_file.pdf]

Supplementary Information

Detecting extracellular G4 DNA motifs in biofilms through energy transfer between DNA-binding dyes TOTO™-1 and SYTO™60

Line Mørkholt Lund[1,2], Gabriel Antonio Salvador Minero[1], Julie Kaysen[1,2], Rikke Louise Meyer[1,3], and Victoria Birkedal[1,2,*]

[1]Interdisciplinary Nanoscience Center (iNANO), Aarhus University, Aarhus, Denmark, [2]Department of Chemistry, Aarhus University, Aarhus, Denmark, and [3]Department of Biology, Aarhus University, Aarhus, Denmark

*To whom correspondence should be addressed (vicb@inano.au.dk)

** Line Mørkholt Lund and Gabriel Antonio Salvador Minero contributed equally.

Table of content

Figure S1: Absorption and emission spectra from SYTO™60 and TOTO™-1 2

Figure S2: Determination of dissociation constants (K_D) 3

Figure S3: Thermal annealing time affects DNA aggregation 4

Figure S4: Fluorescence from TOTO™-1 and SYTO™60 upon TOTO™-1 excitation in KCl-containing buffer 5

Figure S5: Spectral changes at elevated TOTO™-1 concentrations 6

Figure S6: Small FRET effect is observed in cesium-containing reaction buffer 7

Figure S7: Urea-PAGE quantification of DNase I degradation of DNA substrates 8

Figure S8: Examples of fluorescence microscopy images of *S. epidermidis* biofilms stained with immunolabelling 9

Figure S9: KCl and NaCl in TSB media promotes G4 formation in immunolabelled biofilm 10

Figure S10: Fluorescence microscopy images of *S. epidermidis* biofilms stained with SYTO™60 and TOTO™-1 11

Figure S11: Fluorescence microscopy images of *P. aeruginosa* biofilms stained with SYTO™60 and TOTO™-1....12

Figure S12: NMP cannot be used for G4 DNA staining in biofilm 13

Figure S13: Background correction for FRET signal analysis for data presented in Figure 5 14

Figure S14: Hemin addition does not perturb the FRET effect 15

Figure S15: Hemin stabilizes G4-DNA in potassium-containing conditions 16

Figure S16: Different A/D_p ratio thresholding for data presented in Figure 5 17

Figure S17: Effect of nuclease treatment on the FRET signal 18

Table S1: Dye-DNA dissociation constants 19

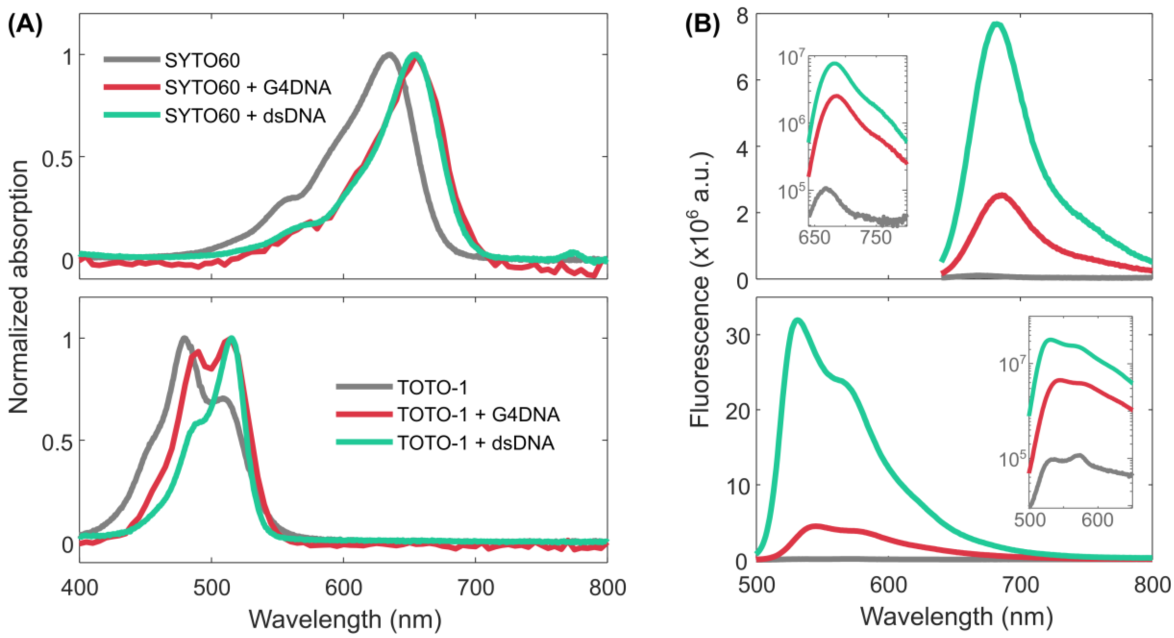

Figure S1: Absorption and emission spectra from SYTO™60 and TOTO™-1. (A) Normalized absorption spectra of SYTO™60 (upper panel) and TOTO™-1 (lower panel) alone as well as in the presence of G4DNA or dsDNA. (B) Fluorescence emission spectra ofSYTO™60 (upper panel) and TOTO™-1 (lower panel) alone as well as in the presence of G4DNA or dsDNA. Fluorescence spectra are shown both with a linear and logarithmic axis to show the very weak fluorescence of unbound dyes.

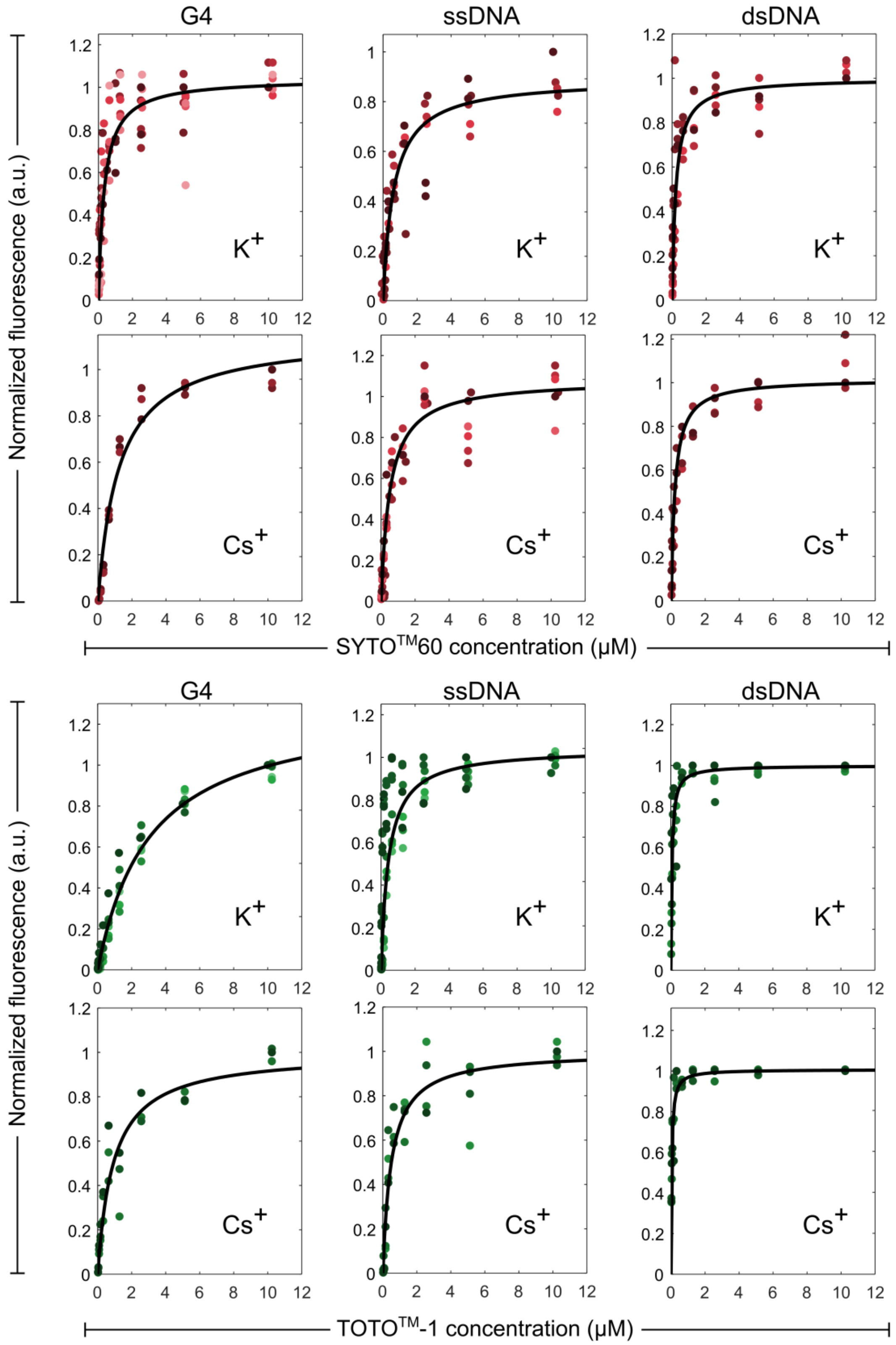

Figure S2: Determination of dissociation constants (*K*D). Plots are shown for SYTO™60 and TOTO™-1 bound to G4 DNA, ssDNA, and dsDNA in potassium- and cesium-containing reaction buffer. Repetitions are shown in different colors. Each measurement series was independently fitted to obtain a K_D value. The resulting average K_D values' binding curves are plotted as black lines, and the average K_D values are found in Table S1.

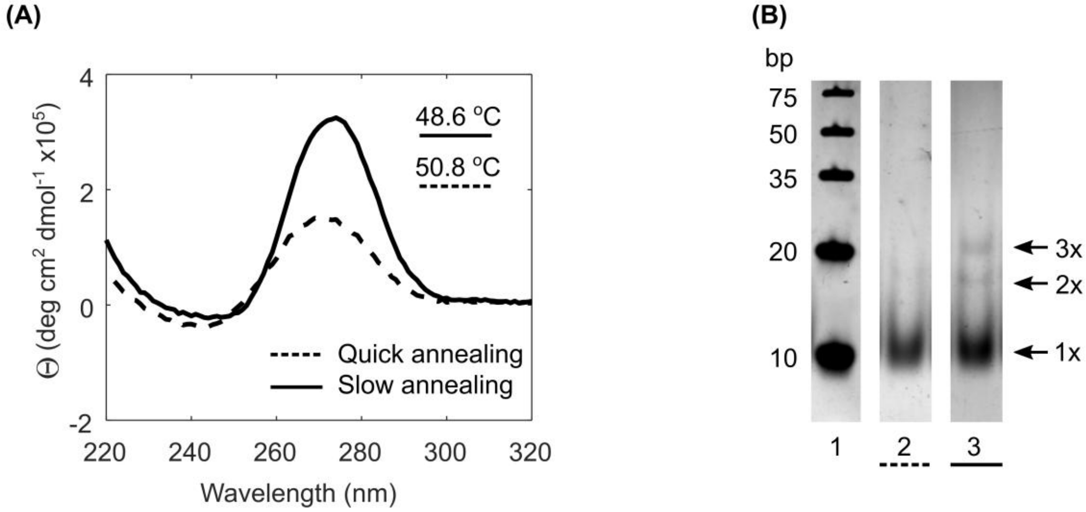

Figure S3: Thermal annealing time affects DNA aggregation. (A) CD spectra of G4DNA at 9°C after quick or slow annealing in 100 mM CsCl buffer, respectively. Here, the quick annealing was performed by heating the sample to 95°C for 5 min followed by a quick cooling to 9°C. The slow annealing was performed as described in the materials and methods as a slow decrease in temperature from 95°C to 9°C overnight. The melting temperatures shown in panel A are similar, however the CD signal is stronger for slow annealed samples, suggesting the presence of aggregates. (B) Native gel (10% 29:1 Acrylamide to Bisacrylamide) with 10 mM CsCl in the gel material showing the migration of 100 ng (28% glycerol) G4 DNA after quick (lane 2) and slow (lane 3) annealing of G4 in 100 mM CsCl buffer. Lane 1 shows a double-stranded DNA ladder. Electrophoresis was performed at 120 V in ½ x TBE for 5 h and stained with SYBR™Gold for 5 min. Higher bands (2x are 3x) are visible for the slow annealing (lane 3), indicating the formation of aggregates. For the slow annealing, higher bands contributed less than 8% to the total intensity in the lane.

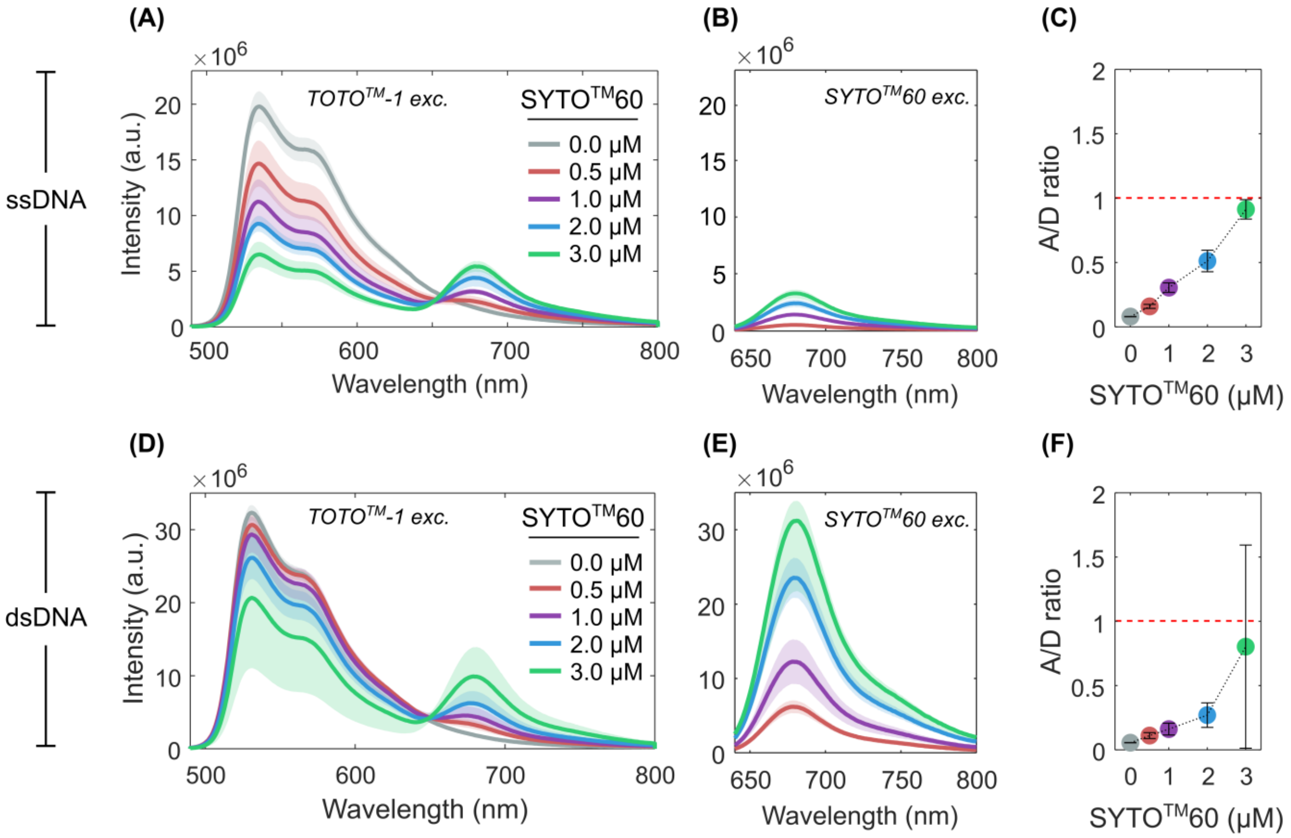

Figure S4: Fluorescence from TOTO™-1 and SYTO™60 upon TOTO™-1 excitation in KCl-containing buffer. Fluorescence spectra upon TOTO™-1 and SYTO™60 excitation and A/D ratios for (A)-(C) ssDNA, and (D)-(E) dsDNA. The samples were all measured at constant DNA and TOTO™-1 concentrations (0.5 μM) and varying SYTO™60 concentrations (0-3 μM) in potassium-containing buffer. For the A/D ratio plots (C) and (F), a dotted line at A/D ratio = 1 is marked for all samples.

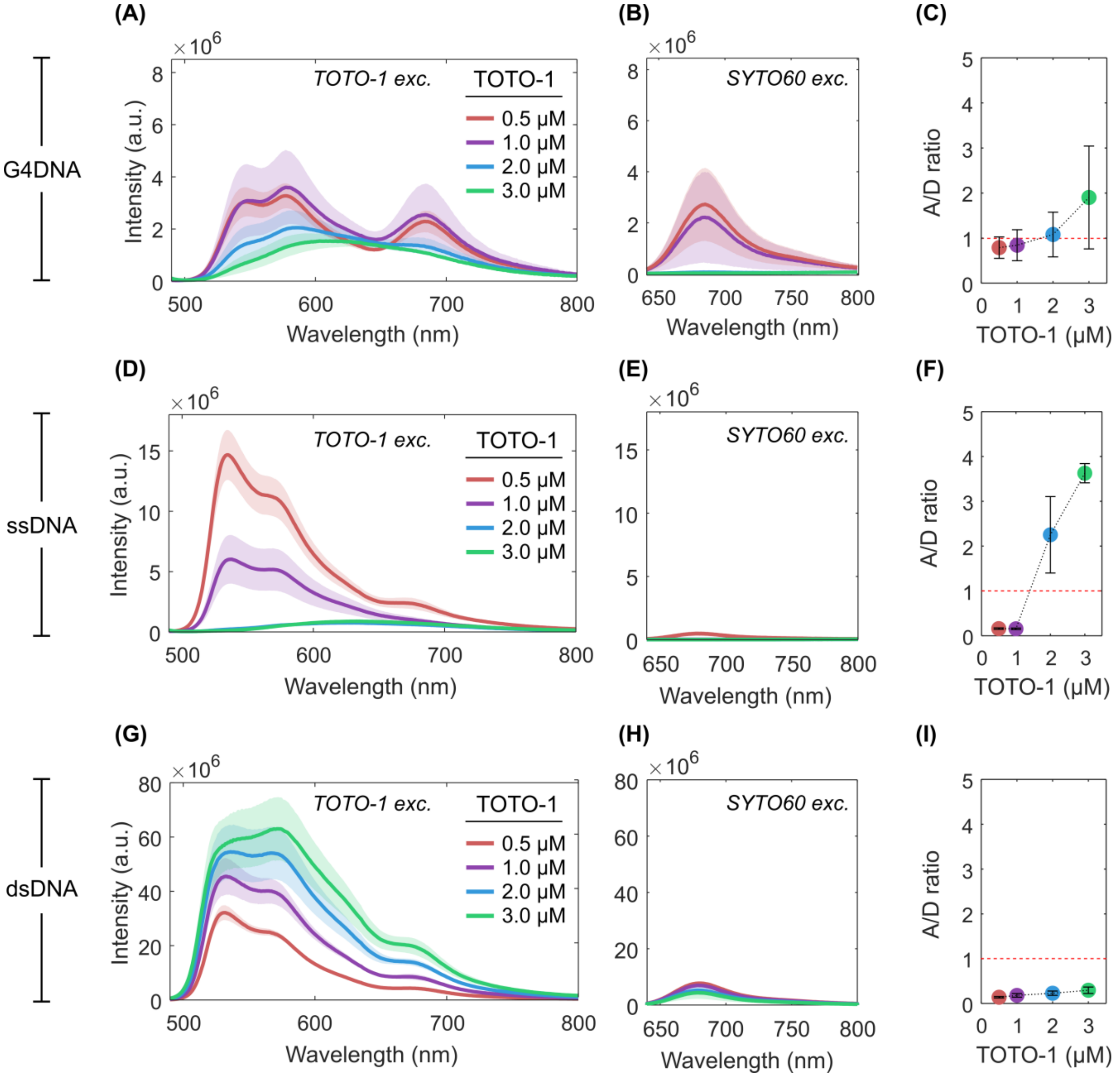

Figure S5: Spectral changes at elevated TOTO™-1 concentrations. Fluorescence spectra upon TOTO™-1 and SYTO™60 excitation, respectively, and A/D ratios for (A)-(C) G4DNA, (D)-(F) ssDNA, and (G)-(I) dsDNA. The samples are all measured at constant DNA and SYTO™60 concentrations (0.5 μM) and varying TOTO™-1 concentrations (0.5-3 μM) in potassium-containing buffer. For the A/D ratio plots (C), (F), and (I), a dotted line at A/D ratio = 1 is marked for all samples.

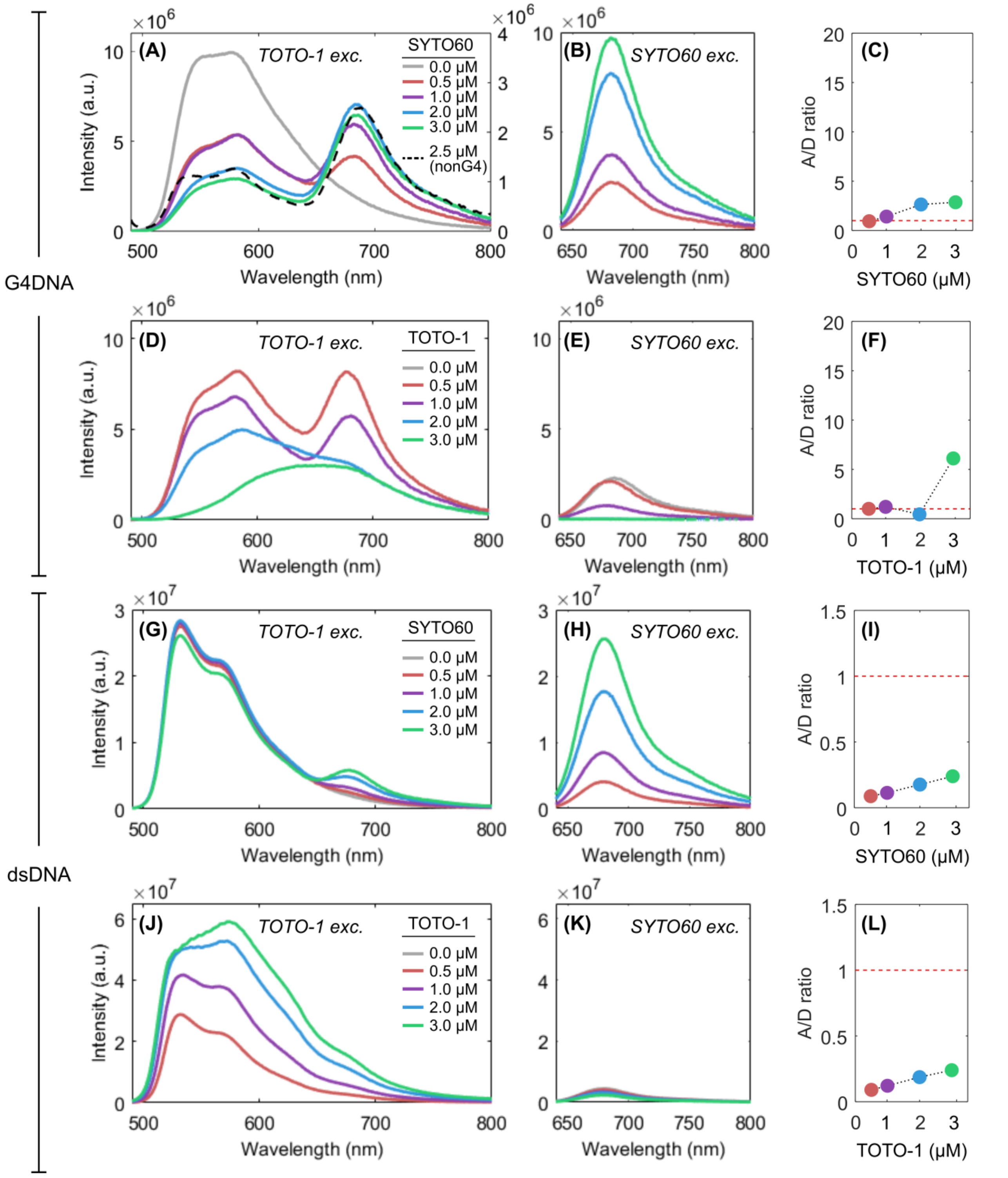

Figure S6: FRET effect is observed in cesium-containing reaction buffer. Fluorescence spectra upon TOTO™-1 and SYTO™60 excitation, respectively for (A)-(F) G4DNA, and (G)-(L) dsDNA in cesium-containing buffer. The DNA and TOTO™-1 concentrations are held constant (0.5 µM) while the SYTO™60 concentration varies (0-3 µM) in (A)-(C) and (G)-(I). The DNA and SYTO™60 concentration is held constant (0.5 µM) while the TOTO™-1 concentration varies (0-3 µM) in (D)-(F) and (J)-(L). There is a FRET signal from G4DNA at elevated SYTO™60 concentration in a cesium-containing buffer (Panel A). Panel (A) also shows the fluorescence spectra from a non-G4 sequence (5'-GGGATGCGACAGAGAGGACGGG-3') with 0.5 µM DNA, 0.5 µM TOTO™-1 and 2.5 µM SYTO™60 in potassium-containing buffer. The y-axis scale for the non-G4 sequence data is shown at the right side. The non-G4 sequence is a G-rich control that does not form G4s. It shows a similar FRET effect as G4DNA in cesium-containing buffer. This suggests that G-rich DNA motifs also produce a FRET signal when unfolded. It is, however, smaller than in the potassium-containing buffer

(Figure 2 in the manuscript), where G4s are folded structures. At elevated TOTO™-1 concentrations, the G4DNA spectra are quenched while dsDNA shows spectral changes. For the A/D ratio plots (C), (F), and (I), a dotted line at A/D ratio = 1 is marked for all samples.

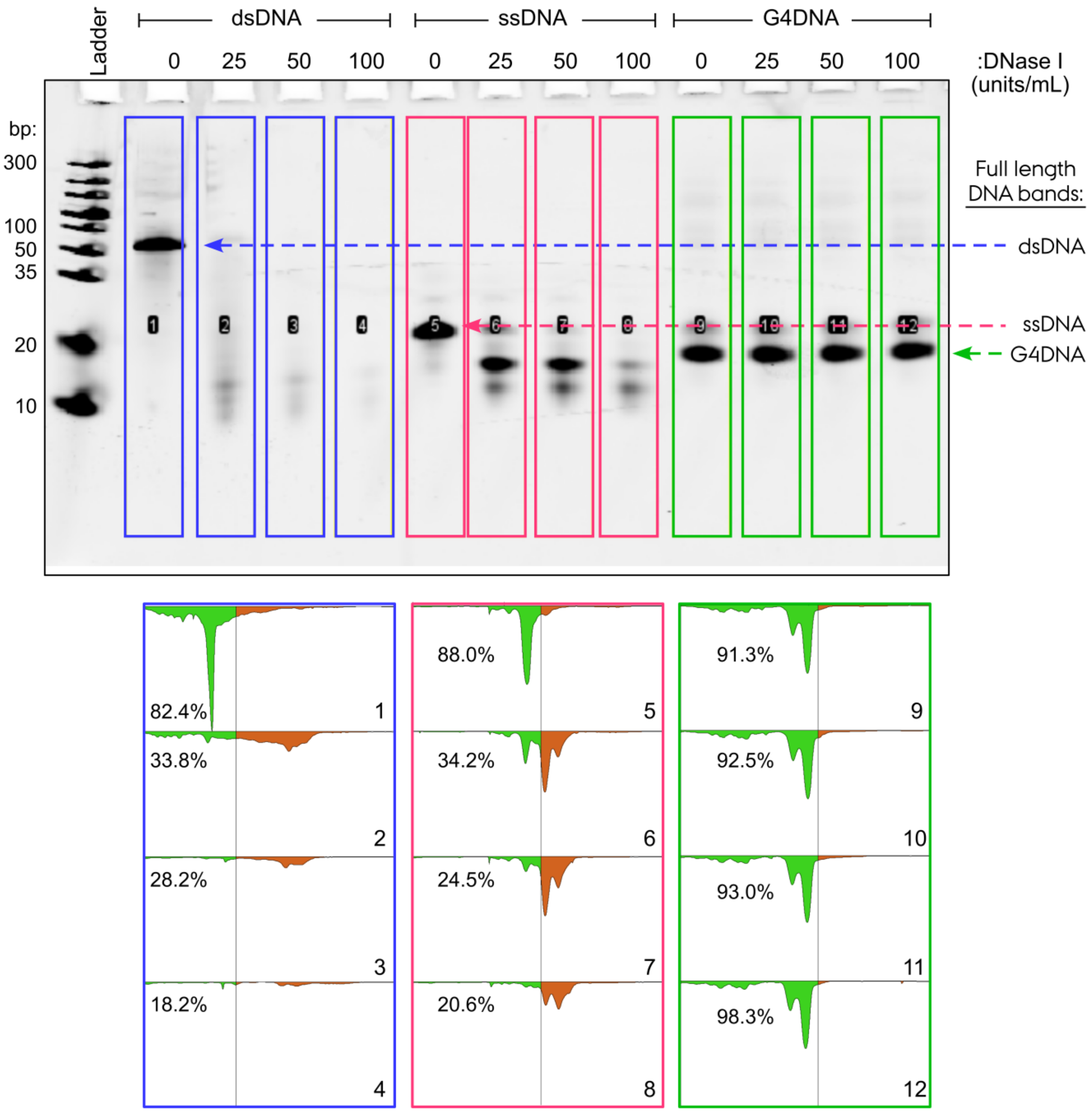

Figure S7: Urea-PAGE quantification of DNase I degradation of DNA substrates. Urea gel (upper panel) and lane profile analysis (lower panel) reveal that DNase I degrades a large portion of the dsDNA and ssDNA substrate (blue and red lanes) while G4DNA is resistant to degradation (green lanes). For G4DNA, it was not possible to denature the G4 fully, since the folded structure is so stable in 100 mM KCl and therefore two bands can be observed in all wells. However, the uncomplete denaturing of G4DNA does not affect the presented results, that G4DNA is resistant to degradation by DNase I.

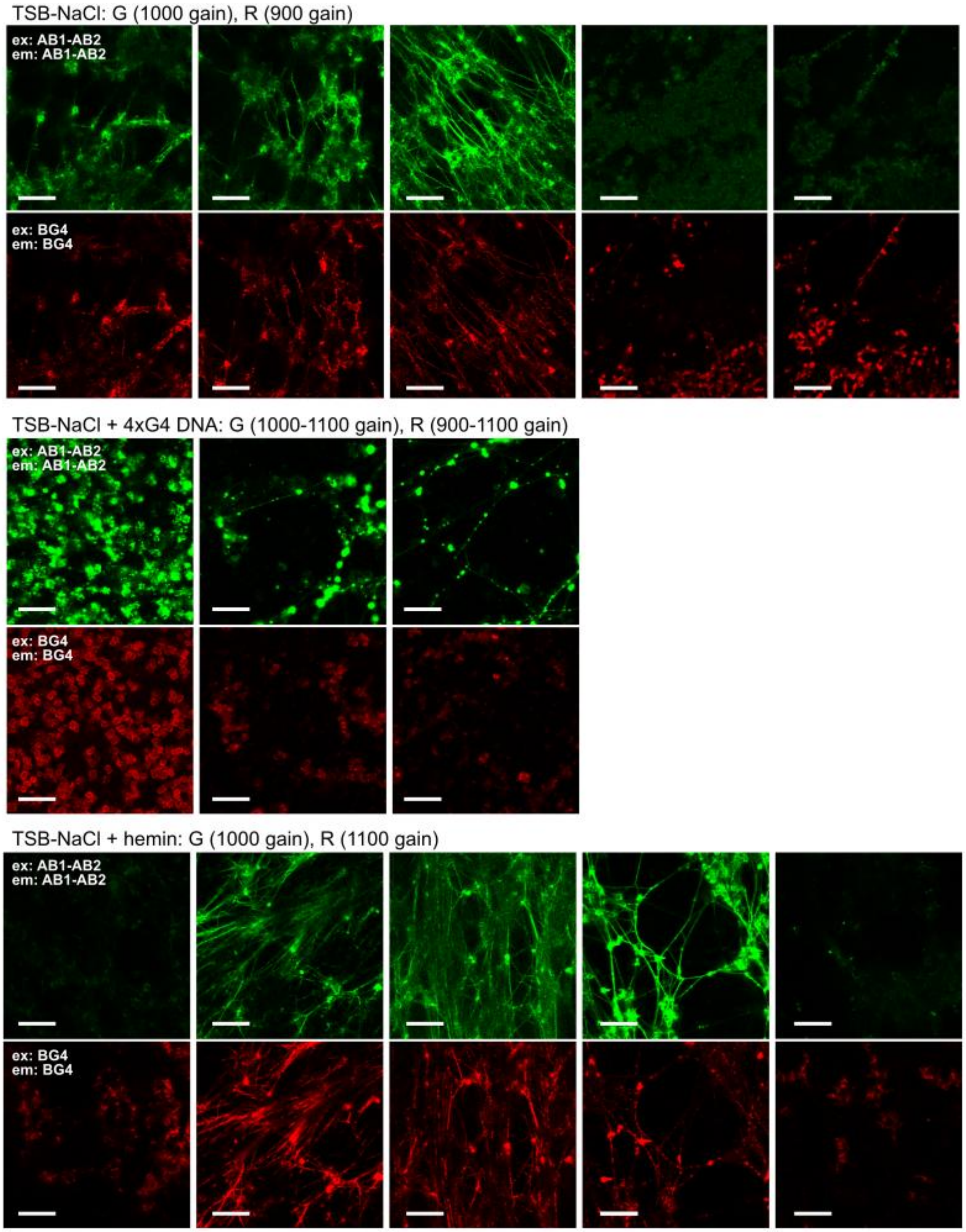

Figure S8: Examples of fluorescence microscopy images of *S. epidermidis* AUH4567 biofilms stained with antibodies as described in Materials and Methods experiment #2. B-DNA appears in green and G4 DNA in red**.** Top panel shows biofilms prepared in the presence of sodium (TSB-NaCl) with the Middle panel shows biofilms with pre-folded G4 DNA added (TSB-NaCl + 4xG4 DNA). Lower panel shows biofilms in the presence of hemin (TSB-NaCl + hemin). Note that different gain settings were used for the various sample conditions as described in the figure text. Scale bar is 10 µm.

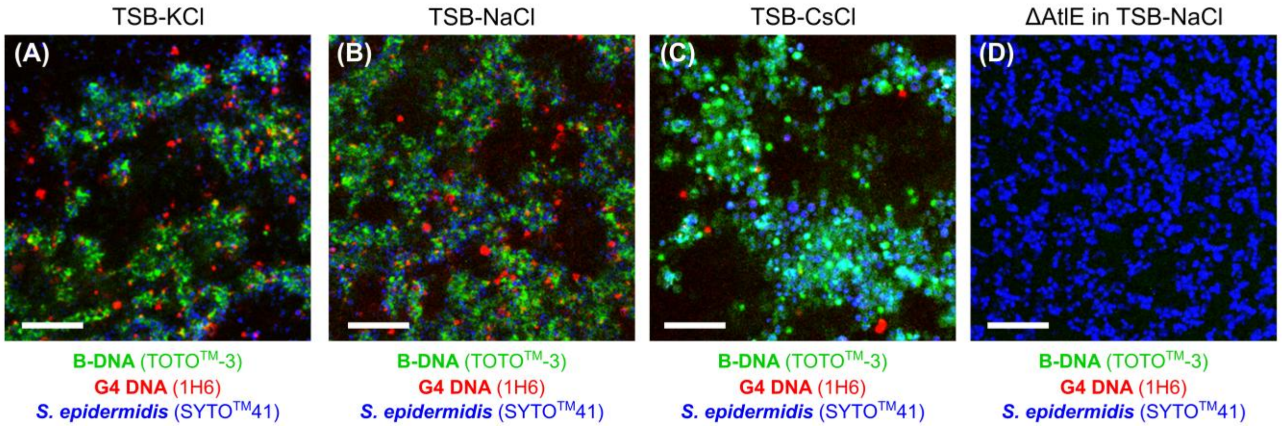

Figure S9: KCl and NaCl in TSB media promotes G4 formation in immunolabelled biofilm. *S. epidermidis* biofilm (strain 1457, grown for 3 days in TSB with 150 rpm shaking) shows extracellular 1H6 signal in the media with (A) KCl and (B) NaCl but not in the media with (C) CsCl. (D) Strain 1457 ΔAtlE biofilm in TSB-NaCl (lacking the autolysin protein necessary for eDNA release) is lacking both eDNA and G4-DNA. Biofilms were immuno-labelled by 1H6 (red) and, subsequently, stained by TOTO™-3 (green) and SYTO™41 (blue) as described in Materials and Methods experiment #3. Scale bar is 10 µm. We observe a greater FRET signal in biofilms grown in both KCl- and NaCl-containing TSB media, which are expected to stabilize G4s, whereas CsCl-containing media produce less.

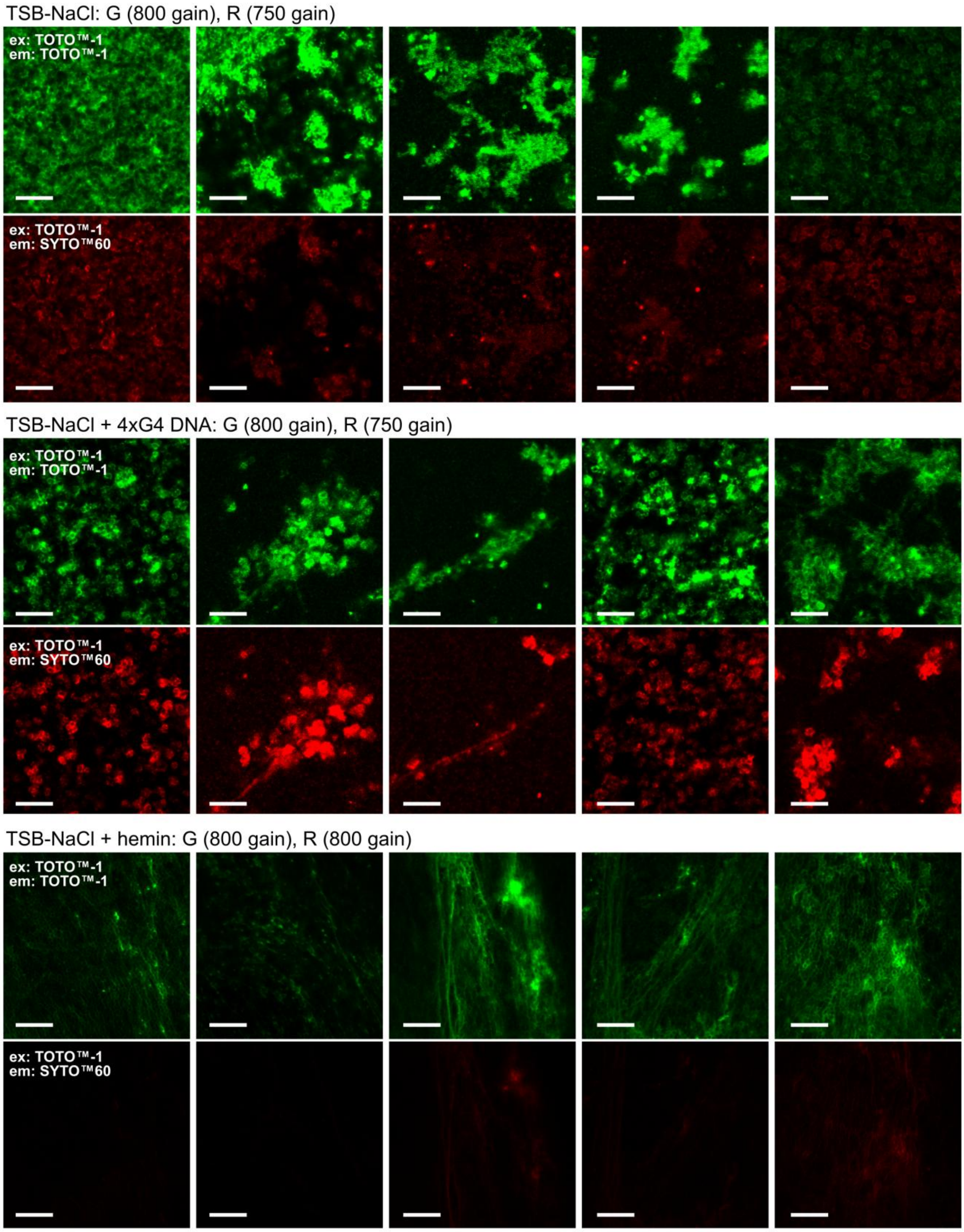

Figure S10: Fluorescence microscopy images of *S. epidermidis* AUH4567 biofilms stained with SYTO™60 and TOTO™-1 as described in Materials and Methods experiment #1. TOTO™-1 (DD) and (DA) signals appear in green and red, respectively. Top panel shows the biofilms prepared in the presence of sodium (TSB-NaCl). Middle panel shows biofilms with pre-folded G4 DNA added (TSB-NaCl + 4xG4 DNA). Lower panel shows biofilms in the presence of hemin (TSB-NaCl + hemin). Note that different gain settings were used for the various sample conditions as described in the figure text. Scale bar is 10 µm. We see a reduction in the signal in the FRET channel when imaging biofilms in the presence of hemin,

likely due to reduced binding of SYTO™60 in the presence of hemin. However, the detected FRET patterns were similar to those obtained with immunolabelling and were not affected by gain settings (Figure 5 and Supplementary Figure S8).

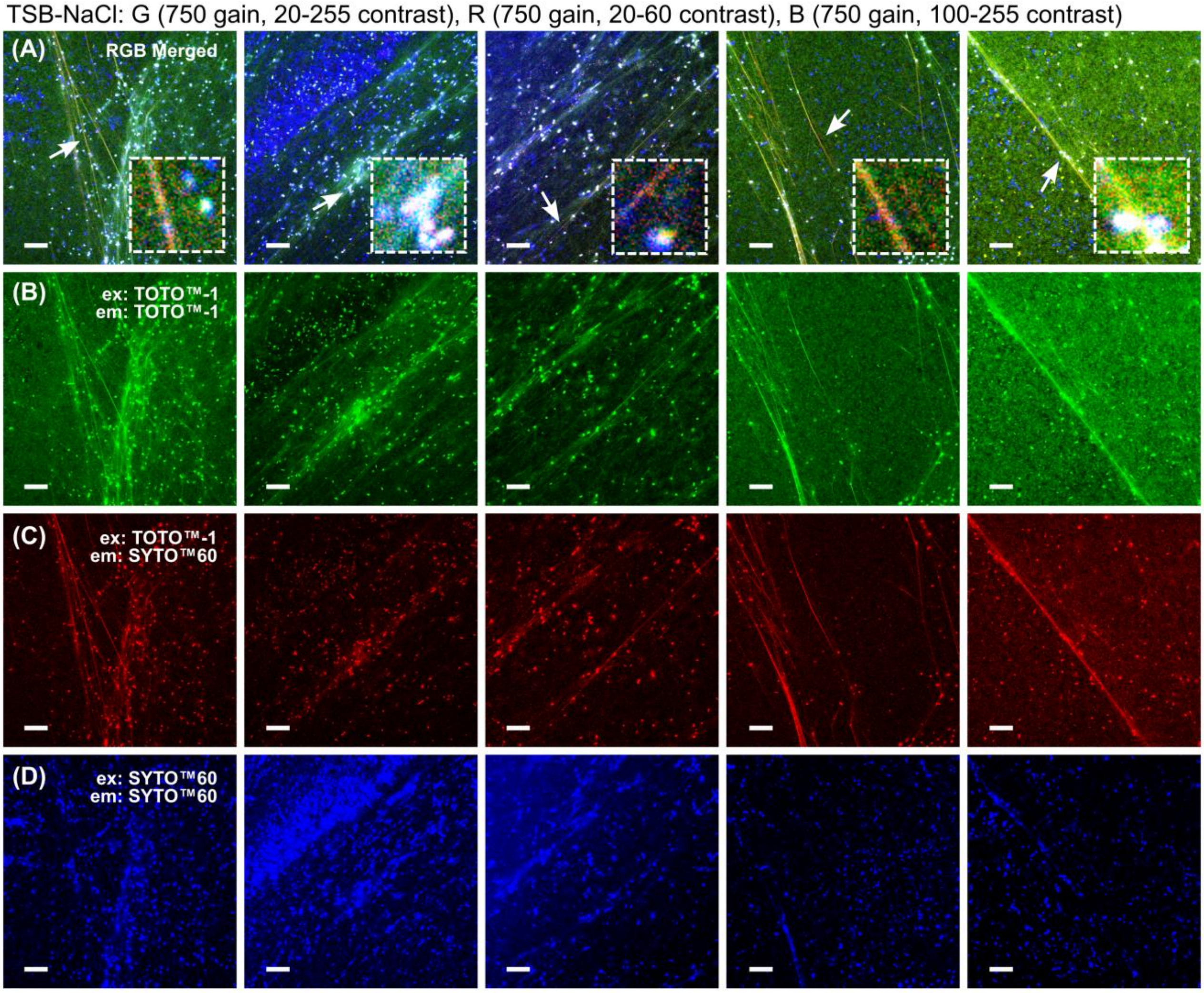

Figure S11: Fluorescence microscopy images of *P. aeruginosa* PAO1 biofilms stained with SYTO™60 and TOTO™-1 as described in Materials and Methods experiment #4. (A) RGB merged images. Scale bar is 10 µm and zoom-in images are 5x5 µm^2. (B) TOTO™-1 emission upon TOTO™-1 excitation shown in green, staining eDNA. Contrast adjusted from 20-255. (C) SYTO™60 emission upon TOTO™-1 excitation, FRET channel, shown in red. Contrast adjusted from 20-60. (D) SYTO™60 emission upon SYTO™60 excitation shown in blue. Contrast adjusted from 100-255. Same imaging settings were used here as described in Materials and Methods, with the difference that the 488 and 639 nm laser power were 0.2% and a gain of 750 was used for all three channels.

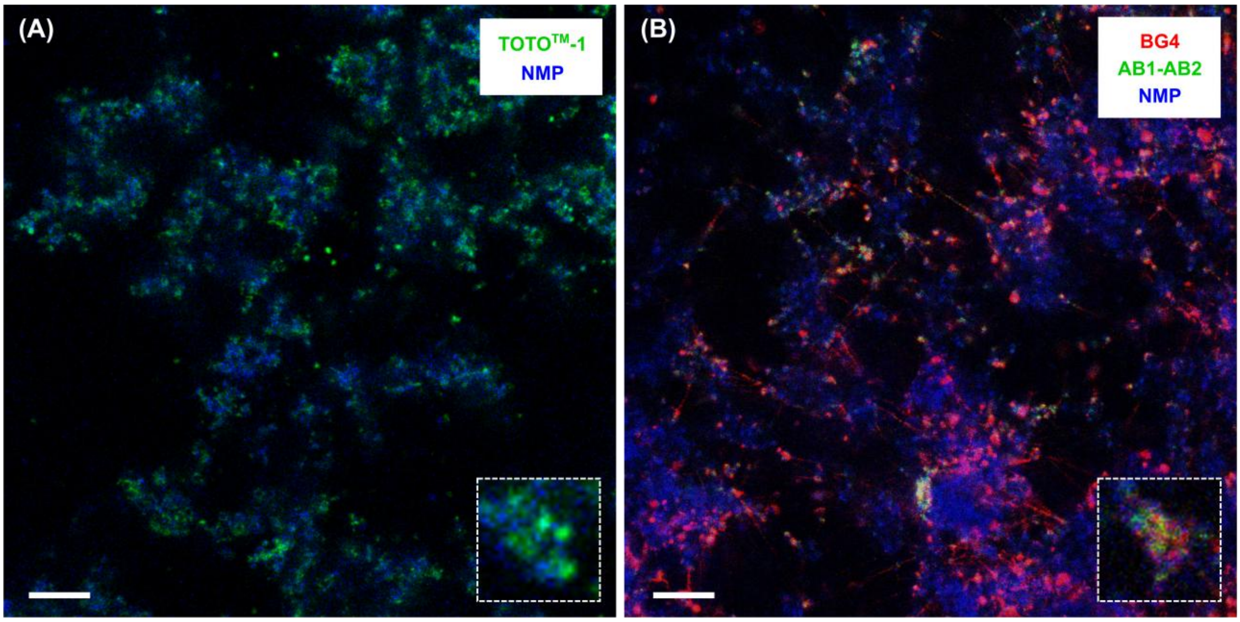

Figure S12: NMP cannot be used for G4 DNA staining in biofilm. *S. epidermidis* 1457 (wild-type) biofilm (3-day in TSB-NaCl with 150 rpm shaking) shows intracellular NMP signal. (A) Biofilm stained by NMP (blue) and TOTO™-1 (green). (B) Biofilm stained by NMP (blue), BG4 (red) and AB1-Ab2 (green). No co-localization of NMP signal with the other extracellular markers was observed. Biofilms were obtained and visualized at the bottom of IbiTreat 96-well plates as described in the Materials and Methods experiment #5. Collectively, the data show that the NMP staining only partially co-localized with BG. However, even in those locations G4s are challenging to see due to the high background from intracellular NMP signal. Scale bar is 10 µm and zoom in images are 5 x 5 μm^2.

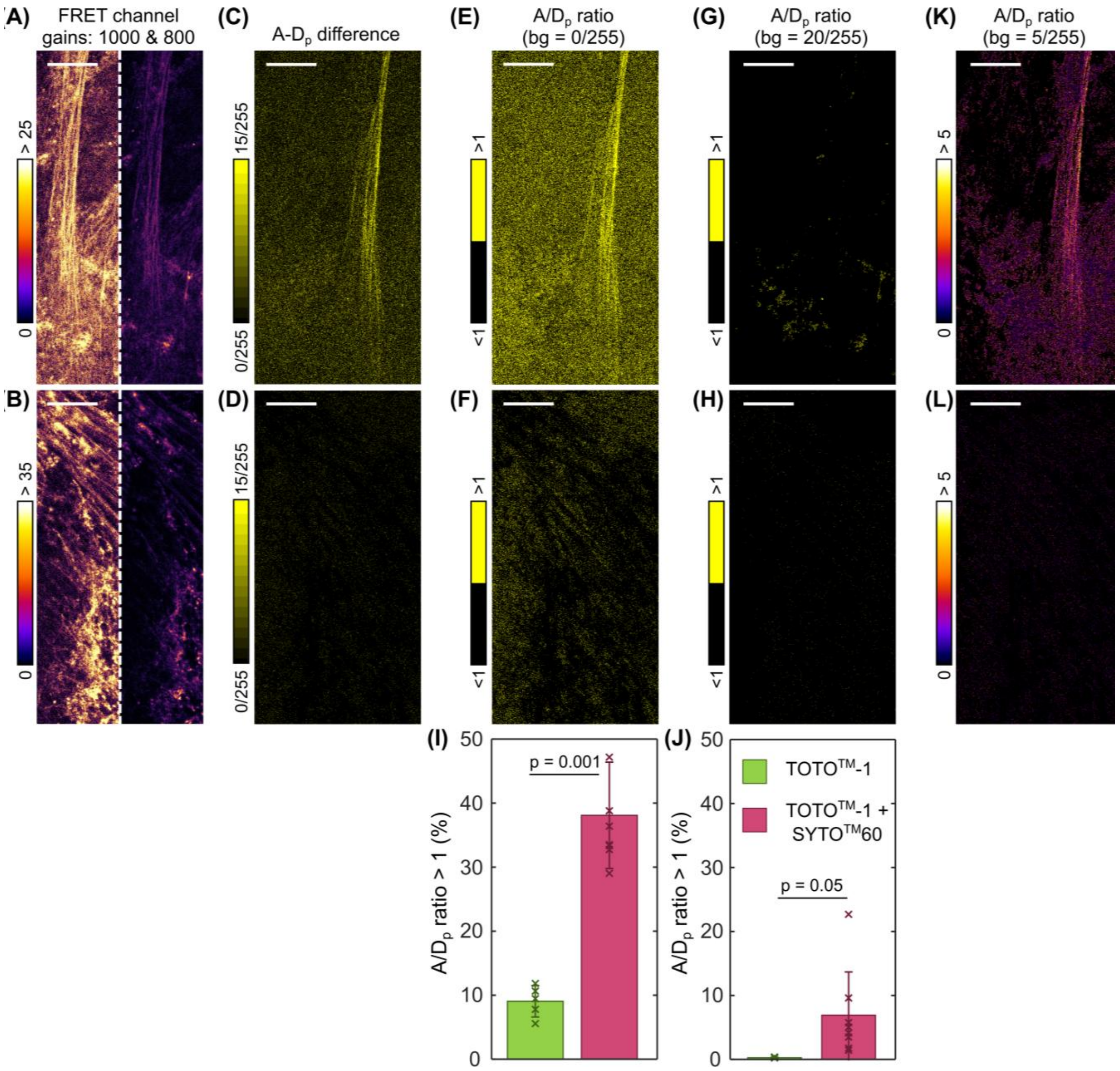

Figure S13: Background correction for FRET signal analysis for data presented in Figure 5. The fluorescence signal in the FRET channel upon TOTO™-1 excitation for gain settings of 1000 (left) and 800 (right) are shown for the biofilm stained with both dyes (A) and TOTO™-1 only (B) as described in Materials and Methods experiment #1. Images show that the same structures are reveled in the biofilms at different gain settings – only the fluorescence intensities are different. Resulting FRET signal from the fluorescence from TOTO™-1 and the FRET signal from biofilms stained either with TOTO™-1 and SYTO™60 or with TOTO™-1 alone. Scale bar for all images is 15 µm. (C-D) Pixel A-D_p difference above zero images for (C) the biofilm stained with both dyes, and (D) the biofilm stained only with TOTO™-1. The pixel A-D_p difference image (no background correction) shows clear areas with FRET signal for the biofilm with both dyes (C), whereas the control biofilm with only TOTO™-1 only shows noise (D). (E-L) Pixel A/D_p ratio images for (E, G, K) the biofilm stained with both dyes, and (F, H, L) the biofilm stained only with TOTO™-1. The pixel A/D_p ratio was determined using background values of zero (E, F) and 20 counts (G-H) and values above one were visualized as yellow pixels. The pixel A/D_p ratio above 1 determined with a background of 5 is shown in Figure 5D. (I and J) The percentage of pixels with A/D_p ratios above one (i.e. number of green pixels with A/D_p ratios above one) were determined for all images stained with both dyes (in red) and with only TOTO™-1 (in green) using a background of zero counts (I), 20 counts (J) and 5 counts (Figure 5E). The p-values from t-tests are indicated. Although the observed trends did not depend on the background chosen in pixel A/D_p ratio images, the clearer images were obtained by adjusting the background intensity to yield structural features similar to those obtained in the corresponding pixel A-D_p difference images. Here, a background value of 5 yields clear A/D_p ratio signatures for the biofilm with both dyes, as well as a significant difference between

the number of pixels with a pixel A/D_p ratio above 1 for the biofilm with both dyes compared to the control. The A/D_p ratio above 1 determined using a background value of 5 is shown in Figure 5D. The corresponding A/D_p ratio images with color gradient are shown for the biofilm stained with both dyes (K) and TOTO™-1 only (L) with black being an A/D_p ratio of zero, and white pixels having an A/D_p ratio above 5.

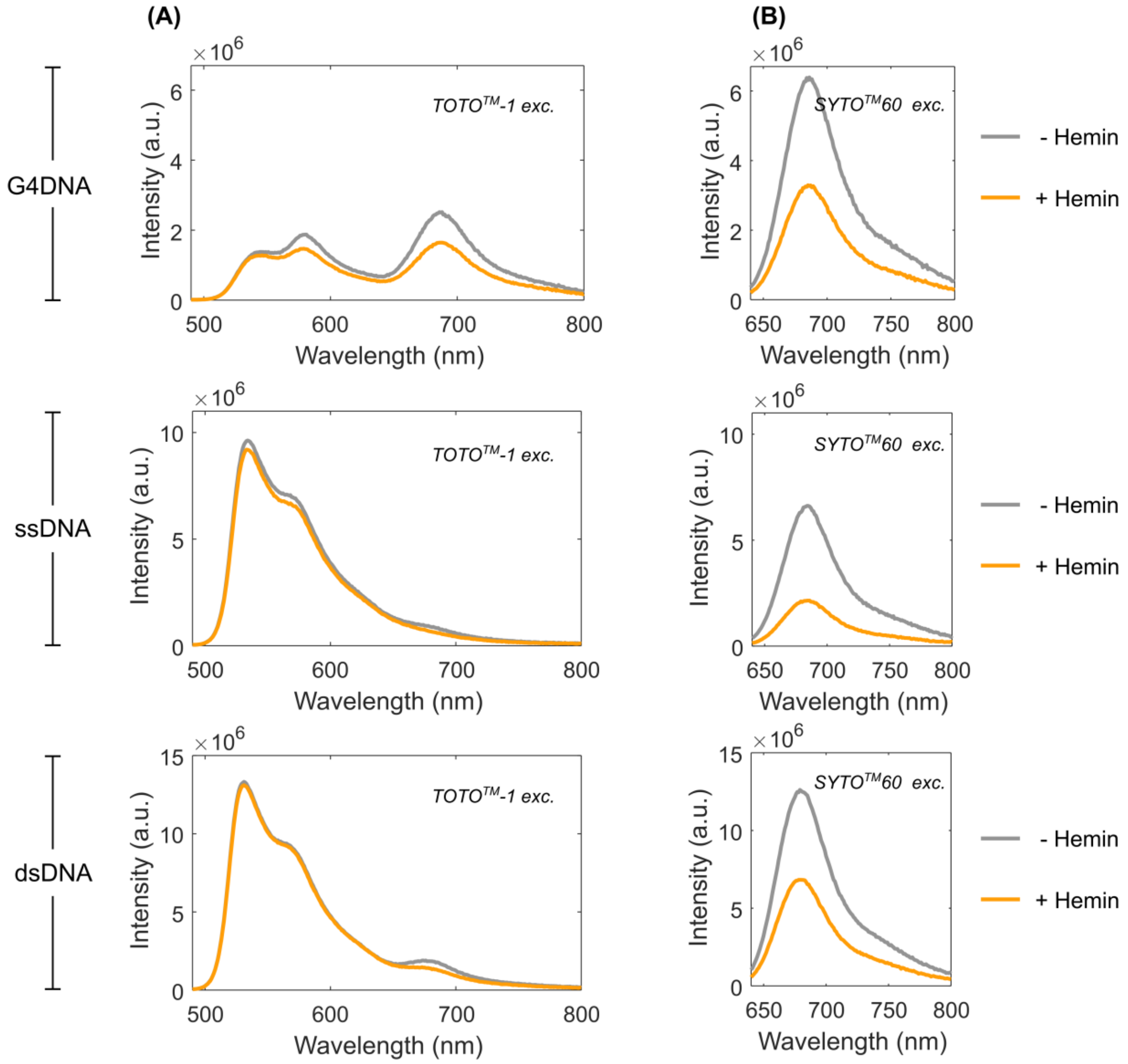

Figure S14: Hemin addition does not perturb the FRET effect. Fluorescence spectra from 0.5 µM TOTO™-1 and 2.5 µM SYTO™60 bound to G4DNA (top), ssDNA (middle), and dsDNA (bottom) upon TOTO™-1 excitation (A) and SYTO™60 excitation (B). In every sample, hemin addition, 0.5 µM, reduces mainly the SYTO™60 fluorescence, thereby decreasing the A/D ratio slightly. Therefore, hemin addition to biofilms is not expected to artificially increase the measured A/D ratio.

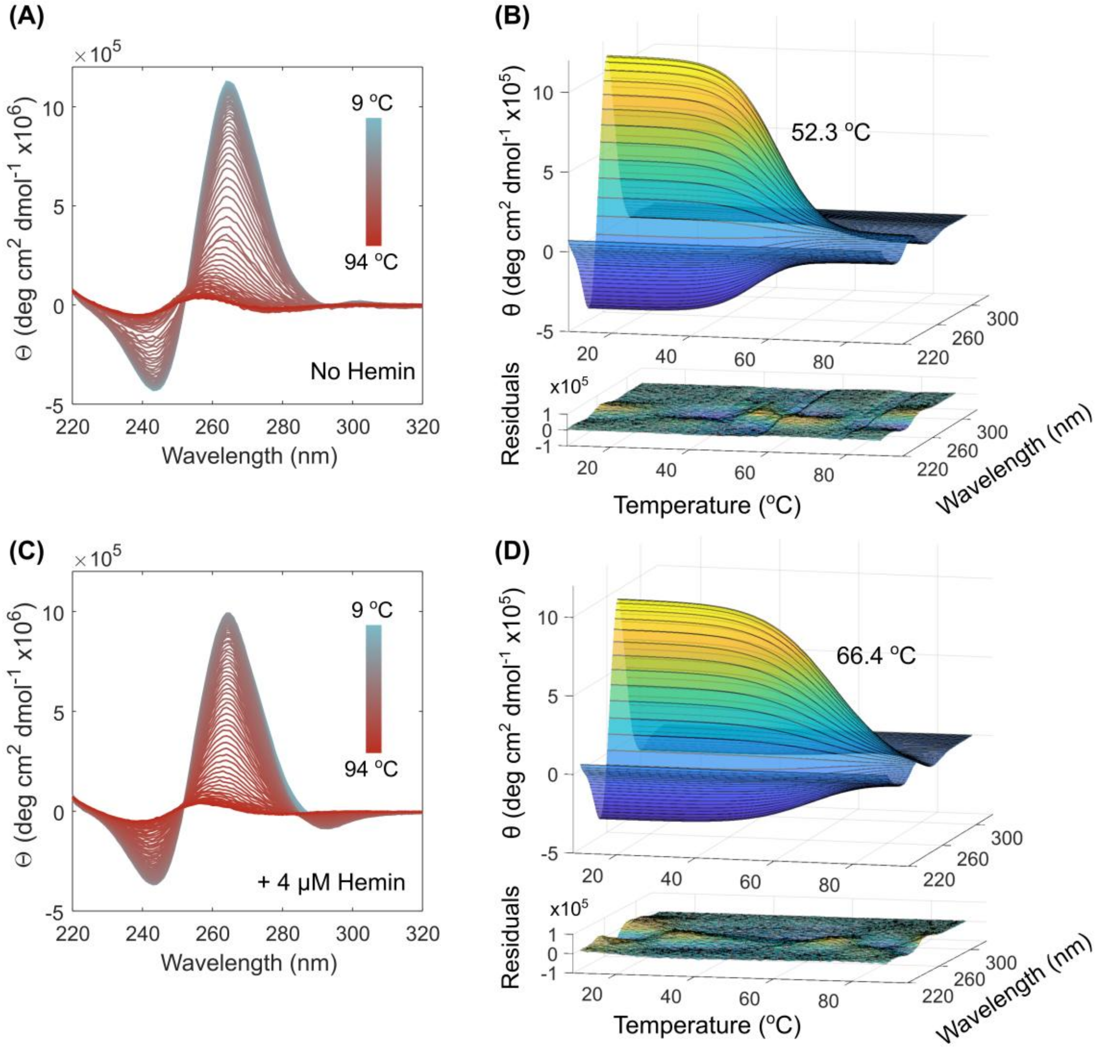

Figure S15: Hemin stabilizes G4DNA in potassium-containing conditions. CD melting results for G4DNA in 10 mM KCl buffer in the absence (A)-(B) and presence (C)-(D) of hemin, respectively. In (A) and (C) the raw CD spectra are shown for temperatures from 9 ^oC to 94 ^oC. (B) and (D) show fit results to the data. The resulting melting temperatures are marked, showing that hemin increases the melting temperature of G4DNA in 10 mM KCl with ~ 14 ^oC.

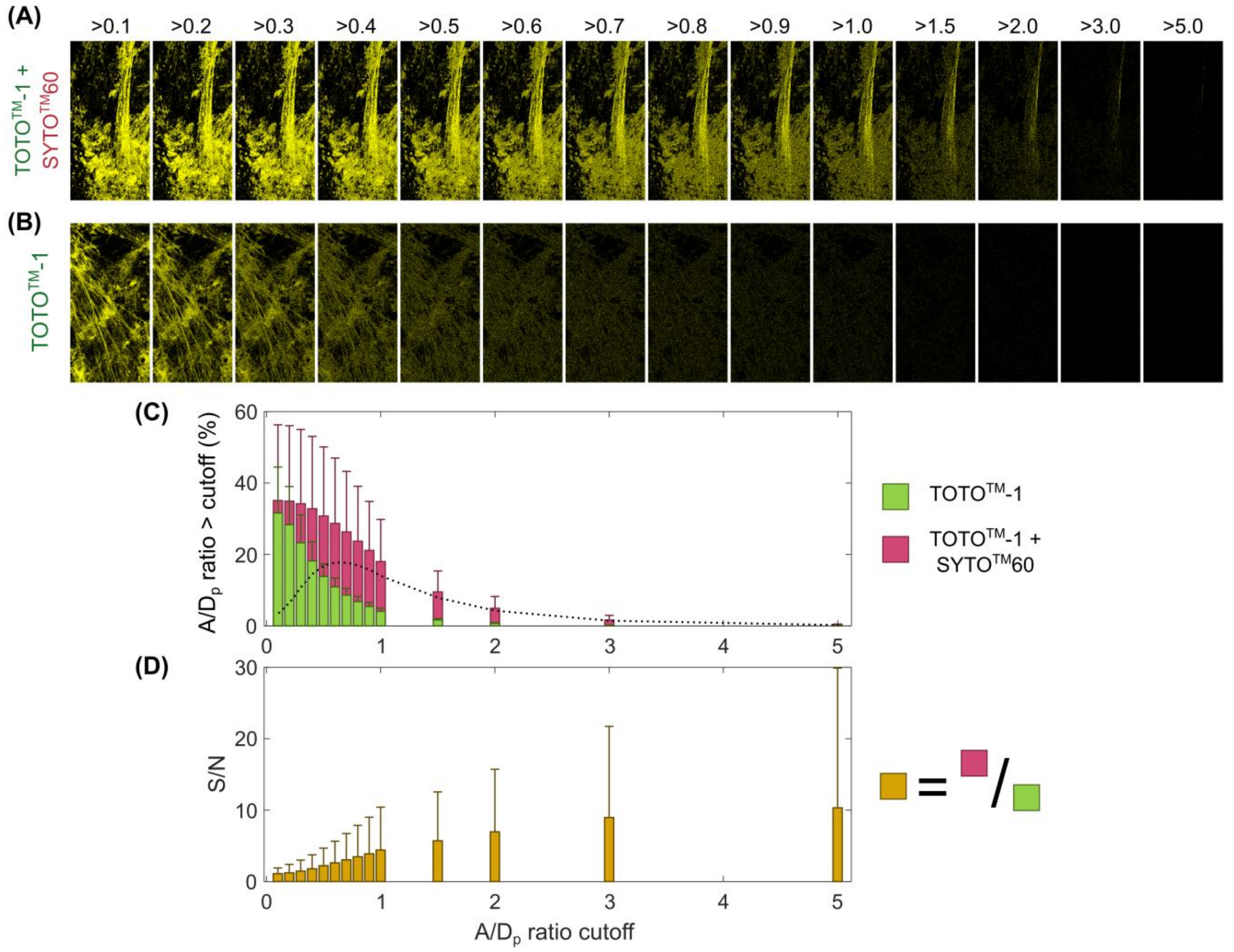

Figure S16: Different A/D_p ratio thresholding for data presented in Figure 5. A/D_p ratio images with different ratio thresholding from 0.1 to 5 for biofilm samples stained in TOTO™-1 in the presence (A) and absence (B) of SYTO™60. (C) Bar plot showing the resulting percentage of pixels with an A/D_p ratio above the threshold for samples in the absence of (green) and presence (red) of SYTO™60. The black dotted line shows the difference between the red and green bars. (D) Resulting signal to noise (S/N) bar plot. The optimal A/D_p ratio cutoff will be in a region with high S/N while also having a significant difference in A/D ratio pixels (black dotted line in (C)).

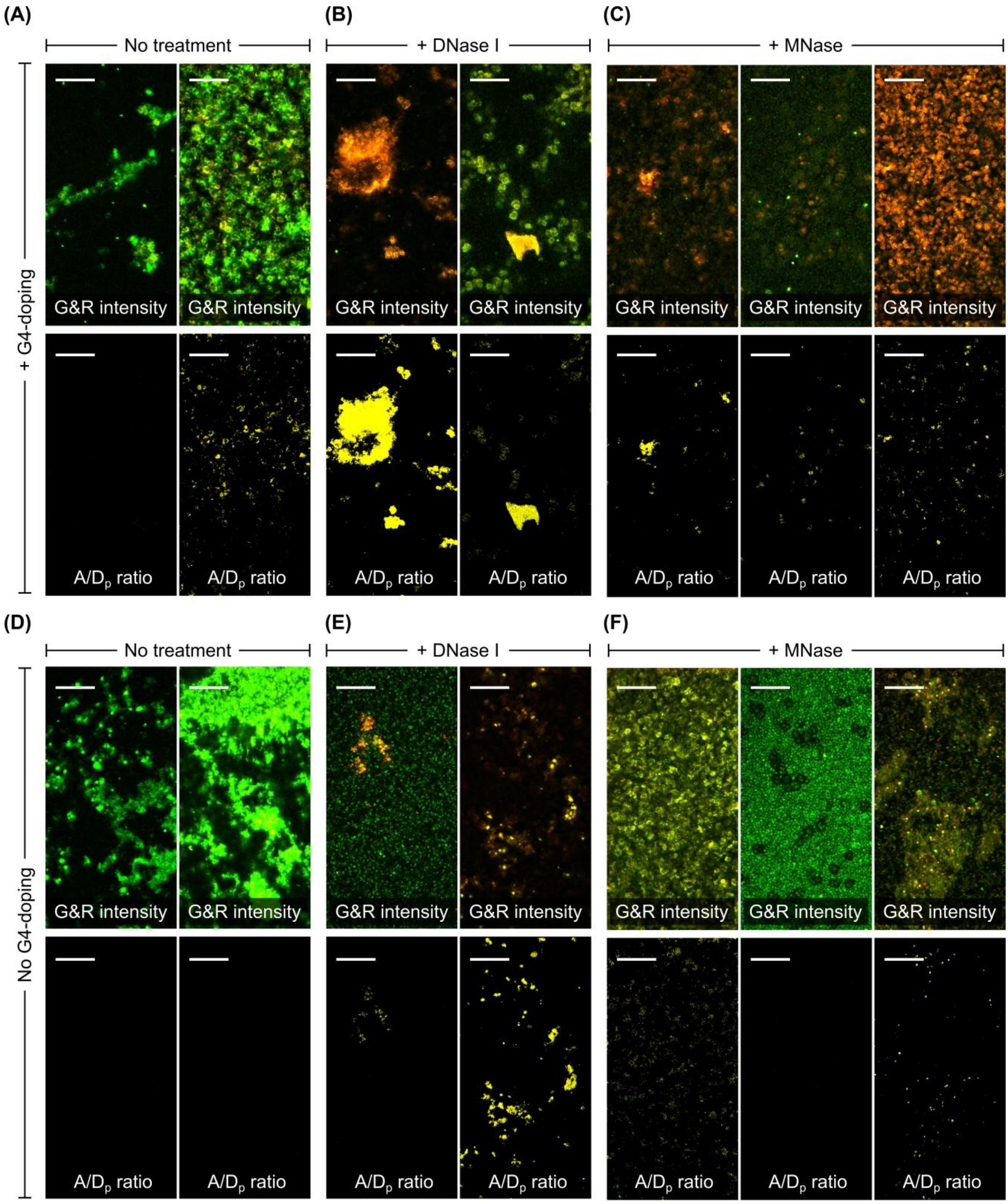

Figure S17: Effect of nuclease treatment on the FRET signal. Examples of 3-day *S. epidermidis* biofilms in TSB-NaCl stained by TOTO™-1 and 12.5 µM SYTO™60 in the presence (A-C) and absence (D-F) of G4-doping using 4xG4DNA. Representative images as described in Methods and Materials experiment #1 are shown for biofilm without treatment (A, D), after DNase I treatment (B, E) and after MNase treatment (C, F). Image overlays are shown of channel 1 (< 630 nm, green, DD) and channel 2 (> 630 nm, red, DA) in the top panels as well as the 'A/D$_p$ ratio above 1' images at the bottom panels. Scale bar = 15 µm.

Table S1: Dye-DNA dissociation constants. Dissociation constants (K_D) found for different dye:DNA combinations in either potassium or cesium-containing buffer conditions. The total number of repetitions is indicated in the last column.

Dye	DNA sequence	Cation type	K_D (µM)	± (µM)	# Repeats
**SYTO™60**	G4DNA	K^+	0.30	0.19	8
		Cs^+	1.24	0.15	3
	ssDNA	K^+	0.62	0.26	5
		Cs^+	0.52	0.15	6
	dsDNA	K^+	0.22	0.19	5
		Cs^+	0.24	0.18	4
**TOTO™-1**	G4DNA	K^+	3.06	1.23	7
		Cs^+	0.92	0.39	3
	ssDNA	K^+	0.44	0.35	6
		Cs^+	0.50	0.16	4
	dsDNA	K^+	0.06	0.04	4
		Cs^+	0.04	0.01	3
